# Proliferation and Apoptosis Adaptor Protein 15 (PEA15), a Potential Oncogenic Regulator of VHL and HIF1A Identified through Proteomic Analysis in Hepatocellular Carcinoma

**DOI:** 10.34133/cancomm.0020

**Published:** 2026-04-08

**Authors:** Yun Seong Jeong, Ji-Hyun Shin, Soo Mi Kim, Bo Hwa Sohn, Sun Young Yim, Ji Hoon Kim, Jae Jun Shim, Sung Hwan Lee, Yun Shin Chun, Sunyoung S. Lee, Hui Dai, Ahmed Kaseb, Koo Jeong Kang, Holger K. Eltzschig, A. Robert MacLeod, Xiaolin Luo, Alexey Revenko, Youngsoo Kim, Ju-Seog Lee

**Affiliations:** ^1^ Department of Systems Biology, The University of Texas MD Anderson Cancer Center, Houston, TX, USA.; ^2^ Department of Physiology, Institute of Medical Science, Jeonbuk National University Medical School, Jeonju, Jeonbuk, Korea.; ^3^ Department of Internal Medicine, Korea University College of Medicine, Seoul, Korea.; ^4^ Department of Internal Medicine, Kyung Hee University Hospital, Seoul, Korea.; ^5^ Division of Hepatobiliary and Pancreas, Department of Surgery, CHA Bundang Medical Center, CHA University, Sungnam, Korea.; ^6^ Department of Surgical Oncology, The University of Texas MD Anderson Cancer Center, Houston, TX, USA.; ^7^ Department of Gastrointestinal Medical Oncology, The University of Texas MD Anderson Cancer Center, Houston, TX, USA.; ^8^ Division of Hepatobiliary and Pancreatic Surgery, Department of Surgery, Keimyung University Dongsan Medical Center, Daegu, Korea.; ^9^ Department of Anesthesiology, Critical Care and Pain Medicine, McGovern Medical School, The University of Texas Health Science Center at Houston, Houston, TX, USA.; ^10^ Ionis Pharmaceuticals, Carlsbad, CA, USA.

## Abstract

**Background:** Hepatocellular carcinoma (HCC) is a leading cause of cancer-related deaths globally. Although the hypoxia-inducible factor 1A (HIF1A) pathway is crucial in HCC progression, its regulatory mechanisms remain unclear as mutations in its primary regulator, von Hippel–Lindau tumor suppressor (*VHL*), are rare in HCC. We aimed to elucidate the role of proliferation and apoptosis adaptor protein 15 (PEA15), identified through proteomic analysis, as a regulator of the VHL/HIF1A pathway and a therapeutic target in HCC. **Methods:** Proteomic and genomic analyses of over 1,000 HCC samples were conducted, identifying *PEA15* amplification. Functional validation involved in vitro and in vivo assays, including gene knockdown, ectopic expression, and antisense oligonucleotide (ASO) therapy in xenograft models. Protein interactions were assessed using immunoprecipitation and ubiquitination assays. **Results:** We identified 3 clinically distinct HCC subtypes and found that *PEA15* was selectively amplified and highly expressed in the mesenchymal (MES) subtype, which exhibited the poorest prognosis. *PEA15* acted as a regulator of the VHL/HIF1A pathway and a key oncogene in HCC. The amplification of *PEA15* was significantly associated with the poor survival of HCC patients. Moreover, by interacting with the β-domain of VHL, PEA15 promoted HCC cell proliferation and migration by inhibiting VHL’s interaction with the VHL/elongin C (ELOC)/elongin B (ELOB)/cullin 2 (CUL2) E3 ligase complex, destabilizing the complex and consequently activating HIF1A. Importantly, pharmacologically inhibiting PEA15 using *PEA15* ASO drugs attenuated tumor burden and restored VHL function in a xenograft mouse model. **Conclusions:** This study identified *PEA15* as a potential oncogene in HCC, regulating the VHL/HIF1A axis and driving tumor progression. Targeting *PEA15* using ASOs offers a promising therapeutic strategy for HCC, particularly in the MES subtype. These findings provide a basis for further exploration of *PEA15*-targeted therapies to improve HCC outcomes.

## Background

Hepatocellular carcinoma (HCC) is one of the most common and deadliest cancers worldwide and is estimated to contribute to more than 700,000 deaths annually [1]. Although several kinase inhibitors have demonstrated a survival benefit for HCC patients [2–4], their impact is modest. Immunotherapy with immune checkpoint inhibitors (ICIs) offers a promising new approach [5–9]. However, clinical outcomes with ICIs remain unsatisfactory, with response rates below 30% owing to HCC tumors’ immunologically cold nature. Thus, there is a clear need for new targeted therapeutic agents for HCC and/or effective strategies to extend the survival of HCC patients. Achieving this goal necessitates a deeper comprehension of the molecular mechanisms driving HCC’s initiation and progression.

During the course of solid tumor progression, cancer cells frequently undergo epithelial-to-mesenchymal transition (EMT), a process in which polarized, immotile epithelial cells transform into migratory mesenchymal cells with increased metastasis and drug resistance [10]. Although EMT is considered a biological feature that promotes poor clinical outcomes in HCC, the molecular and genetic mechanisms governing the acquisition of the mesenchymal phenotype are not fully understood. Hypoxia-inducible factor 1A (HIF1A) is one of the signaling proteins that can provoke EMT by up-regulating EMT-associated genes [11,12], and its expression is increased in HCC tumors and is significantly associated with poor prognosis [13,14]. However, the molecular mechanism of its activation in HCCs has been poorly understood because von Hippel–Lindau tumor suppressor (*VHL*), a frequently mutated upstream regulator of HIF1A in many cancers [15–17], is rarely mutated in HCCs [18]. Therefore, elucidating the mechanisms governing EMT and HIF1A activity in HCC is imperative to impeding tumor progression and improving patient survival.

In this study, we applied large-scale proteomic and genomic profiling of HCC tumors to identify molecular subtypes with distinct clinical outcomes and to search for key oncogenic drivers. Our aim was to discover previously unrecognized pathways and candidate proteins that may contribute to HCC progression and serve as potential therapeutic targets.

## Materials and Methods

### Patients and samples

Archived HCC samples obtained from 113 patients who underwent hepatectomy at The University of Texas MD Anderson Cancer Center (MDACC; Houston, TX, USA) between December 2001 and June 2011 as their primary treatment were used to generate protein expression profile data using a reverse-phase protein array (RPPA) platform. Patients were selected based on the availability of frozen tissue and clinical follow-up data. All samples were frozen in liquid nitrogen and stored at −80 °C until protein extraction. All research was conducted in accordance with both the Declarations of Helsinki and Istanbul and was approved by the MDACC Institutional Review Board (LAB09-0687-MDACC). Of the 113 tumors in the MDACC cohort, 110 were used to generate mRNA expression data using the Illumina microarray platform HumanHT-12 v4.0 Gene Expression BeadChip (accession number GSE134568). Three tumors were excluded due to the low quality of the RNA after purification. Overall survival in the MDACC cohort was calculated from the day of surgery and censored at the end date of follow-up. To test the reproducibility of the proteomic signatures, RPPA data from The Cancer Genome Atlas (TCGA) HCC cohort (*n* = 184) were used.

Gene expression data in the validation cohorts were obtained for HCC patient cohorts from the National Cancer Institute (*n* = 113, GSE4024) [19,20], Korea Research Institute of Bioscience and Biotechnology (*n* = 100, GSE16757) [21], Samsung Medical Center (*n* = 240, GSE36376) [22], and University of Modena and Reggio Emilia (*n* = 78, GSE54236) [23] through the National Center for Biotechnology Information (NCBI) Gene Expression Omnibus (GEO) database (https://www.ncbi.nlm.nih.gov/geo/). Additional data from Zhongshan Hospital (*n* = 159) were accessible through the National Omics Data Encyclopedia (https://www.biosino.org/node/home) (accession number OEP000321), which previously identified 3 proteomic subtypes (ZS-S1, ZS-S2, and ZS-S3) [24]. These 5 cohorts were used as the validation cohorts for mRNA signatures later. Genomic and proteomic data (*n* = 371) from a TCGA study [18] were analyzed using the University of California, Santa Cruz, Xena tool (https://xena.ucsc.edu/) for validation of proteomic signatures and association of proteomic subtypes with genomic features. Copy number amplification data of proliferation and apoptosis adaptor protein 15 (*PEA15)* from TCGA pan-cancer cohort (*n* = 10,713) were obtained from cBioportal (https://www.cbioportal.org/).

Patients or the public were not involved in the design, or conduct, or reporting, or dissemination plans of our research.

### Proteomic data from RPPAs

RPPA experiments were performed with the 113 surgically removed frozen HCC samples from the MDACC cohort and 184 HCC samples from TCGA cohorts described previously [25,26]. Briefly, protein was extracted from tumors using an RPPA lysis buffer [1% Triton X-100, 50 nM Hepes, pH 7.4, 150 nM NaCl, 1.5 nM MgCl_2_, 1 mM EGTA, 100 nM NaF, 10 nM sodium pyrophosphate, 10% glycerol, 1 nM phenylmethylsulfonyl fluoride (PMSF), 1 nM Na_3_VO_4_, 10 μg/ml aprotinin]. Lysis buffer was used to lyse frozen tumors with a Precellys homogenizer (Bertin Instruments). Tumor lysates were adjusted to a concentration of 1 μg/μl as determined using a bicinchoninic acid assay and boiled with 1% sodium dodecyl sulfate. Tumor lysates were manually diluted in 5-fold serial dilutions with lysis buffer. An Aushon Biosystems 2470 arrayer typically printed 1,056 samples of tumor lysates on nitrocellulose-coated slides (Grace Bio-Labs). Slides were probed with 201 validated primary antibodies (Table S1), followed by corresponding secondary antibodies [goat anti-rabbit immunoglobulin G (IgG), goat anti-mouse IgG, or rabbit anti-goat IgG]. RPPA data were processed and normalized as described previously [25–28]. In total, 201 antibodies were used to test the 113 samples from the MDACC cohort and 184 samples from the TCGA cohort. All samples were processed and printed together in the same batch.

### Gene expression data for human tissues

Gene expression data from the MDACC cohort were obtained using the HumanHT-12 v4.0 Gene Expression BeadChip. Initially, total RNA was extracted from fresh frozen HCC samples employing a mirVana RNA isolation and labeling kit (Ambion). Subsequently, 500 ng of total RNA underwent labeling and hybridization following the Ambion’s protocols. Following the scanning of bead chips using an Illumina BeadArray Reader, the microarray data underwent normalization using the quantile normalization method implemented in the Linear Models for Microarray Data package within the R language environment [29]. Prior to further analysis, the expression level for each gene was transformed into a logarithmic base of 2. The primary microarray data for these human HCC samples are publicly available in the GEO database under accession number GSE134568.

### Data analysis

For analysis of proteomic and transcriptomic data, ConsensusClusterPlus R package (v.3.12) [30] and The BRB-ArrayTools (National Cancer Institute) [31] were used. Heatmaps of protein and mRNA expression patterns were generated using the Cluster and TreeView software programs [32]. To find the optimal number of clusters, we evaluated *k* values ranging from 2 to 7 using consensus clustering with 100 resampled datasets. The optimal number of clusters was defined as the one with the smallest slope of curve decline in the abscissa range of 0.1 to 0.9 in consensus cumulative distribution function (CDF) plots. We selected *k* = 3 as the optimal number of subtypes according to the consensus CDF and delta plot.

Two different statistical cutoff values were used in the analysis of expression data, as the number of variables differed in the protein and mRNA expression datasets. HCC prognoses were estimated using Kaplan–Meier plots and the log-rank test. Heatmaps of protein and mRNA expression patterns were generated using the Cluster and TreeView software programs [32].

For the selection of subtype-specific mRNA sets, mRNA expression data from the MDACC (*n* = 110) were used. Briefly, multiple 2-sample *t* tests for all possible combinations of the 3 subtypes were performed. Gene expression differences were considered statistically significant if the *P* value was less than 0.001 and there was at least a 2-fold difference between the compared subtypes. Genes with significant differences in 2 possible comparisons were considered to be subtype-specific genes. A total of 613 mRNAs were identified, and all gene expression data from the training and validation cohorts were normalized to *z* scores. To identify a class of individual patients in validation cohorts, a previously developed approach was used [33,34]. Briefly, protein and mRNA expression data in the training set (protein signature or mRNA signature in the MDACC cohort) were combined to form a classifier according to a Bayesian compound covariate predictor [35] in the BRB-ArraysTools. The robustness of the classifier was estimated using a misclassification rate determined during leave-one-out cross-validation for the training set. After the Bayesian compound covariate predictor classifier was used to dichotomize the patients according to the signatures, the prognostic significance of the subtypes was estimated using Kaplan–Meier plots (log-rank test). Gene network analysis was performed with Ingenuity Pathway Analysis (IPA) as described in the QIAGEN IPA user manual. The significance of the enriched upstream regulator genes was estimated using the Fisher exact test.

### In vivo tumorigenesis assay

All mouse experiments were approved by and performed in accordance with the guidelines of the Institutional Animal Care and Use Committee (IACUC; 1802-RN02) at the MDACC. All mice were housed in a pathogen-free facility under 12-h light/dark cycle. Mice were monitored daily for signs of distress, including weight loss, lethargy, or tumor burden. The humane endpoint was determined as a 20% weight loss, severe lethargy, or tumor reaching ≤1.5 cm in diameter. Euthanasia was performed using a 70% CO_2_ chamber followed by cervical dislocation.

For an in vivo tumorigenesis assay, control or PEA15-depleted HLF cells (5 × 10^6^) and mock or stably PEA15-overexpressing HepG2 cells (1 × 10^7^) were mixed with Matrigel (1:1) and subcutaneously injected into 8-week-old NCr nude mice (Charles River Laboratories). Tumor sizes were then measured weekly, and tumor volumes were determined using the standard formula *L* × *W*^2^ × 0.52, where *L* is the longest diameter and *W* is the shortest diameter. For evaluating the effect of *PEA15* antisense oligonucleotide (ASO) treatment on HCC, 6-week-old NCr nude mice were injected with HLF cells (5 × 10^6^) and then after 2 weeks randomly divided into 4 groups: phosphate-buffered saline (PBS), control ASO, PEA15 ASO1, and PEA15 ASO2. ASO treatments were performed by intraperitoneal injections, 3 times a week at 50 mg/kg for 4 weeks. Tumor growth and volume were assessed using caliper measurements weekly.

### Cell lines and cell culture

HepG2, PLC/PRF/5, SK-Hep1, HEK-293T, SNU-387, SNU-449, and SNU-475 were obtained from the American Type Culture Collection (ATCC). FOCUS was a gift from J. R. Wands at Brown University Medical School (Providence). JHH5, JHH7, HUH-7, and HLF cells were obtained from the Health Science Research Resources Bank (Osaka, Japan). LO2 cells were obtained from Cell Bank of Chinese Academy of Sciences (Shanghai, China). The hepatoblastoma cell line HepG2, the human HCC cell lines HUH-7, PLC/PRF/5, JHH7, LO2, HLF, SK-Hep1, SNU-475, and FOCUS, and the human embryonic kidney cell line HEK-293T were cultured in Dulbecco’s modified Eagle’s medium supplemented with 10% (v/v) fetal bovine serum (FBS; Sigma-Aldrich) and 1% (v/v) penicillin–streptomycin (Gendepot). SNU-387, SNU-449, and SNU-475 cells were cultured in RPMI 1640 medium (Corning) supplemented with 10% (v/v) FBS and 1% (v/v) penicillin–streptomycin. JHH5 cells were cultured in William’s E medium (Sigma-Aldrich) supplemented with 10% (v/v) FBS, 2 mM l-glutamine (Gendepot), and 1% (v/v) penicillin–streptomycin. All cell lines were cultured at 37 °C with CO_2_ in a humidified incubator.

### Colony formation assay

Cells (1 × 10^3^ per well) were plated into a 12-well plate and cultured for 10 d. The complete culture medium was changed every 2 d. Cell colonies were stained with crystal violet after methanol fixation and quantitated using the ImageJ software program (National Institutes of Health). The experiment was performed in triplicate, and the data were analyzed using Prism 10 software.

### Viral transduction

For lentiviral transduction, pLKO-short hairpin RNA (shRNA) of green fluorescent protein (shGFP), pLKO-shScramble, pLKO-shPEA15, pCDH, pCDH-PEA15, or pCDH-HIF1A plasmids were transfected with the packaging plasmids psPAX2 and pCMV-VSV-G into HEK-293T cells for 2 d, and filtered viral particles were used to infect target cells. After 48 h of transduction, cells were selected in medium containing 2 μg/ml puromycin (Gibco) for pLKO-shRNA and pCDH-puro-PEA15 clones or 10 μg/ml blasticidin (Gibco) for pCDH-blasticidin-PEA15 clones until noninfected cells died. pLKO-shRNA vectors were obtained from the Sigma-Aldrich MISSION shRNA library. The shRNA target sequences were as follows: shPEA15-1, 5​′-C​CGG​TAC​TGG​CAG​TGC​CTG​GTT​TAG​CTC​GAG​CTA​AAC​CAG​GCA​CTG​CCAGTATTTTTG-3′; shPEA15-2, 5​′-C​CGG​GCA​GAA​TCA​AAT​TGC​TAC​ATA​CTC​GAG​TAT​GTA​GCA​ATT​TGA​TTCTGCTTTTTG-3′; shPEA15-3, 5​′-C​CGG​CCT​CTC​CTA​CAT​TGA​GCA​CAT​CTC​GAG​ATG​TGC​TCA​ATG​TAG​GAGAGGTTTTTG-3′; shGFP, 5′-CAAGCTGACCCTGAAGTTCAT-3′; and shRNA specific to Scramble, 5​′-C​CTA​AGG​TTA​AGT​CGC​CCT​CGC​TCG​AGC​GAG​GGC​GAC​TTAACCTTAGG-3′. shGFP and shRNA specific to scramble were used as controls.

### Inhibitor-based treatments

PLC/PRF/5 or HepG2 cells were treated with the de novo protein synthesis inhibitor cycloheximide (C4859, Sigma-Aldrich) or proteasome inhibitor MG132 (M7449, Sigma-Aldrich). Cycloheximide (10 μg/ml) was mixed with complete culture medium and then incubated for the indicated periods. MG132 (10 μM) was mixed with complete culture medium and then incubated for 4 h before harvesting cells for Western blotting (WB) analysis.

### WB and immunoprecipitation

For WB, the following antibodies were used: anti-PEA15 (1:500, #13091, Cell Signaling Technology), anti-PEA15 (1:500, sc-166678, Santa Cruz Biotechnology), anti-phospho-PEA15 (Ser^104^, 1:1,000, #2776, Cell Signaling Technology), anti-phospho-PEA15 (Ser^116^, 1:1,000, #44-836G, Invitrogen), anti-VHL (1: 5,000, sc-135657, Santa Cruz Biotechnology), anti-β-actin (1:5,000, sc-4778, Santa Cruz Biotechnology), anti-α-tubulin (1:5,000, sc-32293, Santa Cruz Biotechnology), anti-HIF1A (1:500, #610958, BD Biosciences), anti-prolyl hydroxylase domain containing protein 2 (PHD2) (1:1,000, sc-271835, Santa Cruz Biotechnology), anti-factor inhibiting HIF-1 (FIH1) (1:1,000, sc-365128, Santa Cruz Biotechnology), anti-glutathione *S*-transferase (GST) (1:5,000, sc-138, Santa Cruz Biotechnology), anti-hemagglutinin tag (HA) (1:5,000, #901513, BioLegend), anti-histidine (His) probe (1:1,000, sc-8036, Santa Cruz Biotechnology), anti-elongin B (ELOB) (1:1,000, #629302, BioLegend), anti-elongin C (ELOC) (1:1,000, #613101, BioLegend), anti-cullin 2 (1:1,000, #51-1800, Invitrogen), anti-ring box 1 (RBX1) (1:1,000, 11922, Cell Signaling Technology), anti-Snail family transcriptional repressor 1 (SNAI1) (1:1,000, #3879, Cell Signaling Technology), anti-signal transducer and activator of transcription 3 (STAT3) (1:1,000, #9139, Cell Signaling Technology), anti-SMAD family member 3 (SMAD3) (1:1,000, #9523, Cell Signaling Technology), and anti-vascular endothelial growth factor A (VEGFA) (1:1,000, #50661, Cell Signaling Technology). Horseradish peroxidase-linked anti-rabbit IgG (7074S, Cell Signaling Technology), horseradish peroxidase-linked anti-mouse IgG (7076S, Cell Signaling Technology), Mouse TrueBlot ULTRA (18-8817-33, Rockland Immunochemicals), and Rabbit TrueBlot (18-8816-33, Rockland Immunochemicals) were used as secondary antibodies for WB. For WB with HCC cell lysates, whole-cell lysates were prepared in radioimmunoprecipitation assay (RIPA) buffer (1% Triton X-100, 10 mM tris–HCl, pH 8.0, 0.1% sodium dodecyl sulfate, 0.1% sodium deoxycholate, 140 mM NaCl, 1 mM EDTA, 0.5 mM EGTA) containing phosphatase inhibitors (P3200, GenDEPOT) and protease inhibitors (P3100, GenDEPOT). After centrifugation at 20,000*g* for 15 min at 4 °C, proteins in lysates were quantified and processed for WB with antibodies. For WB with tissue lysates, paired tumor and adjacent normal liver tissue lysates were prepared in lysis buffer (1% Triton X-100, 50 mM Hepes, pH 7.4, 150 mM NaCl, 1.5 mM MgCl_2_, 1 mM EGTA, 10% glycerol, 100 mM NaF, 10 mM sodium pyrophosphate, 1 mM Na_3_VO_4_) containing phosphatase inhibitors and protease inhibitors. A Precellys 2 mL Soft Tissue Homogenizing Ceramic Beads Kit (10011152, Cayman Chemical) was used to homogenize tumors and normal tissue according to the manufacturer’s protocol. After centrifugation at 20,000*g* for 15 min at 4 °C, the supernatant was quantified and processed for WB with antibodies. For immunoprecipitation (IP), whole-cell lysates were prepared in IP buffer [25 mM tris–HCl, pH 7.4, 150 mM NaCl, 1 mM EDTA, 1% Nonidet P-40 (NP-40), 5% glycerol] containing phosphatase inhibitors and protease inhibitors. After centrifugation at 20,000*g* for 20 min at 4 °C, the supernatant was quantified, and 1 to 2 mg of protein in lysates were processed for IP with anti-HA-conjugated agarose beads (26181, Thermo Fisher Scientific).

### In vivo ubiquitination assay

For an Ni-nitrilotriacetic acid (NTA) pull-down assay, HEK-293T cells were cotransfected with the His-Ubiquitin, PEA15 or His-Ubiquitin, HA-VHL, PEA15 plasmids for 24 to 48 h, and PBS-washed cells were lysed with denaturing buffer A (6 M guanidine–HCl, 0.1 M Na_2_HPO_4_/NaH_2_PO_4_, 10 mM imidazole, pH 8.0) and sonicated. The cell extracts were then incubated with Ni-NTA beads (R90101, Invitrogen) for 3 h at room temperature. The beads were washed with Buffer T1 (25 mM tris–HCl, 20 mM imidazole, pH 6.8) and Buffer A/T1 mixture. WB of precipitated proteins was then performed with the indicated antibodies.

### Cell viability analysis

Cells (2 × 10^3^ per well) were plated into a 96-well plate and cultured for 4 d. Their viability was measured using Cell Proliferation Kit II (11465015001, Roche) or Cell Counting Kit-8 (CK04, Dojindo Molecular Technologies) according to the manufacturer’s instructions. This experiment was performed in triplicate, and the data were analyzed using Prism 10 software (GraphPad Software). For PEA15 ASO treatments, HLF cells (2 × 10^3^ per well) were plated into a 96-well plate and treated with control or PEA15 ASOs, then cultured for 4 d. Their viability was measured using Cell Counting Kit-8 according to the manufacturer’s instructions.

### Migration assay

Cells (1 × 10^5^) were suspended in serum-free medium and then seeded on an 8-μm-pore Transwell membrane insert (353097, Corning) in a 24-well plate. Complete culture medium containing 10% FBS was placed on the bottom part of the plate. After 6 h of seeding for HLF and SNU-475 cells and 24 h for HepG2 and FOCUS cells at 37 °C in 5% CO_2_, cells adherent to the upper part of the membrane were wiped off using a cotton applicator. Cells were then washed with PBS 3 times and stained with crystal violet after methanol fixation. Migrated cells were quantitated using the ImageJ program. The experiment was performed in triplicate, and the data were analyzed using Prism 10 software.

### Quantitative reverse transcription polymerase chain reaction

Total RNA was extracted from FOCUS cells using an RNeasy Plus Mini Kit (74134, QIAGEN) according to the manufacturer’s instructions. One microgram of RNA was reversely transcribed using iScript Reverse Transcription Supermix (1708840, Bio-Rad), and quantitative reverse transcription polymerase chain reaction (qRT-PCR) was performed using SsoAdvanced Universal SYBR Green Supermix (1725272, Bio-Rad) and an Eppendorf RealPlex4 system (Eppendorf). The thermal cycling condition included an initial denaturation phase at 95 °C for 30 s, followed by 40 cycles including denaturation at 95 °C for 10 s, annealing at 60 °C for 30 s, and extension at 95 °C for 15 s. The fold changes in the relative quantification of mRNAs were calculated using the comparative Ct method. Glyceraldehyde-3-phosphate dehydrogenase (*GAPDH*) was used for normalization, and the data were analyzed using Prism 10. For qRT-PCR, the following primers were used: PEA15 forward, 5′-CTAGGGGAGGGGGCTGAGTT-3′; PEA15 reverse, 5′-GGTGGGGGTTGAGTGGTCTC-3′; HIF1A forward, 5′-GAACGTCGAAAAGAAAAGTCTCG-3′; HIF1A reverse, 5′-CCTTATCAAGATGCGAACTCACA-3′; VHL forward, 5′-AGAGCGATGCCTCCAGGTT-3′; VHL reverse, 5′-TGACGATGTCCAGTCTCCTGTAA-3′; GAPDH forward, 5′-CGCTGAGTACGTCGTGGAGTC-3′; GAPDH reverse, 5′-GCAGGAGGCATTGCTGATGA-3′; solute carrier family 2 member 1 (SLC2A1) forward: 5′-TGCAGGCTTCTCCAACTGGAC-3′; SLC2A1 reverse: 5′-GTCGGGATTCCTAGAGAGTCC-3′; insulin-like growth factor binding protein 3 (IGFBP3) forward: 5′-CGCTACAAAGTTGACTACGAGTC-3′; IGFBP3 reverse: 5′-CTATTCATACCCGTCGGAGAGG-3′; plasminogen activator inhibitor 1 (PAI-1) forward: 5′-CTCATCAGCCACTGGAAAGGC-3′; PAI-1 reverse: 5′-AGGTGTCCACTCAGACCGAGT-3′; fibronectin 1 (FN1) forward: 5′-CAACACCGAGGTGACTGAGAC-3′; FN1 reverse: 5′-GTTTCACCACTGTGGTAACAGAG-3′; VEGFC forward: 5′-GCCAATCACACTTCCTGCCGA-3′; and VEGFC reverse: 5′-CTAAGGTACTGTAGACACCTGG-3′.

### DNA constructs

Protein expression vectors pCDH-PEA15-Puro, pCDH-PEA15, pCDH-PEA15 (Δ1–28aa), and pCDH-PEA15 (Δ1–54aa) were generated by cloning PCR-amplified PEA15 cDNA into the *Nhe*I and *Not*I or *EcoR*I and *Bam*HI restriction enzyme sites of a pCDH-EF1-MCS-T2A-Puro vector. pcDNA3-HA-PEA15 (*PEA15* wild-type) and pcDNA3-HA-PEA15 S104A S116A (PEA15-AA) plasmids were provided by N. Ueno at MDACC. *PEA15* and PEA15-AA were cloned into pENTR3C and subsequently cloned into pCDH destination vectors (pCDH-PEA15-blasticidin) using a Gateway cloning system (Invitrogen). GST-PEA15, GST-PEA15 NT (1–81aa), GST-PEA15 CT (82–130aa), GST-PEA15 H1234 (1–54aa), GST-PEA15 H56 (55–81aa), GST-PEA15 H123 (1–39aa), GST-PEA15 H12 (1–28aa), GST-PEA15 H23 (16–39aa), GST-PEA15 H234 (16–54aa), GST-PEA15 H34 (29–54aa), and GST-PEA15 H3456 (29–81aa) were generated by cloning the PCR-amplified *PEA15* cDNA into the *Bam*H1 and *Sal*1 restriction enzyme sites of a pGEX-6P-3 vector. HA-HIF1A-pcDNA3 (Addgene plasmid 18949) was a gift from W. Kaelin at the Dana-Farber Cancer Institute. For an HIF1A rescue experiment, pCDH-HIF1A-blasticidin was cloned from HA-HIF1A-pcDNA3 using the Gateway cloning system. pcDNA HA-VHL, pcDNA HA-VHL beta domain (1–154aa), pcDNA HA-VHL (Δ95–123aa), pGEX-4T-1 VHL, pGEX-4T-1 VHL beta domain (1–154aa), and pGEX-4T-1 VHL alpha domain (155–213aa) were gifts from D. S. Min at Yonsei University.

### Recombinant protein production

Expression of GST-fusion proteins was induced in *Escherichia coli* Rosetta2 (DE3; Sigma-Aldrich) cells. After 3 h of induction of their expression with 0.5 mM isopropyl β-d-1-thiogalactopyranoside (IPTG) (R1171, Thermo Scientific) at 37 °C in lysogeny broth (LB) medium (46-050-CM, Corning), cells were harvested and resuspended in lysis buffer (50 mM tris–HCl, pH 7.5, 150 mM NaCl, 0.1% NP-40, 1 mM PMSF) containing protease inhibitors followed by sonication. GST-fusion proteins were purified on Glutathione Sepharose 4B beads (17-0756-01, GE Healthcare) according to the manufacturer’s instructions.

### GST pull-down assay

For the GST-PEA15 pull-down assay, 1 to 5 μg of GST-PEA15 proteins were incubated with the same amount of recombinant His-VHL (TP760451, OriGene Technologies) in binding buffer (50 mM tris–HCl, pH 7.5, 150 mM NaCl, 0.1% NP-40, 1 mM PMSF) at 4 °C for 4 h to overnight. The resulting protein complex was pulled down with Glutathione Sepharose 4B beads, washed 4 times with binding buffer, and subjected to WB. The GST-VHL pull-down assay procedures were the same as those for the GST-PEA15 pull-down assay except that GST-VHL proteins were incubated with recombinant PEA15 protein (ab87717, Abcam).

### ASOs

All ASOs used in this study were 16 nucleotides in length, connected sequentially by phosphorothioate internucleoside linkages [36]. At both the 5′ and 3′ ends, the 3 nucleotides were composed of 2′−4′ constrained 2′-O-ethyl (cEt)-modified ribonucleotides. These modifications enhanced the ASOs’ affinity to the target mRNA and improved their resistance to cellular exo- and endonucleases. The central portion of the ASOs was made up of 10 deoxynucleotides, which enabled recognition and cleavage of the target mRNA by ribonuclease (RNase) H1 in the ASO:RNA duplex. To identify ASOs that are effective in reducing *PEA15* mRNA, we performed a systematic screen of a library consisting of 300 ASOs with distinct target sequences, following a previously established methodology [37]. Briefly, transfections were carried out using Lipofectamine 2000 reagent (11668027, Thermo Fisher Scientific) according to the manufacturer’s instructions. Approximately 1.0 × 10^5^ HLF liver cancer cells per well were seeded in 6-well plates 24 h prior to transfection. ASOs (50 to 100 nM) were combined with Lipofectamine 2000 (10 μl/ml) in Opti-MEM medium and incubated under the conditions recommended by the manufacturer. After 5 h, the transfection mixture was replaced with fresh complete medium. Cells were collected 24 to 48 h post-transfection for RNA analysis, and *PEA15* expression was quantified using Bio-Rad’s Droplet Digital PCR (ddPCR) system following the manufacturer’s protocol. This screening led to the identification of the 5 most effective ASOs. The sequences of best ASOs were as follows: control ASO (ASO ID: 792169): 5′-CGCCGATAAGGTACAC-3′; PEA15 ASO1 (ASO ID: 1235289): 5′-CAACTTAGTGGTAGCC-3′; PEA15 ASO2 (ASO ID: 1235214): 5′-ATCTAGTAAAGAAGGG-3′; PEA15 ASO3 (ASO ID: 1235156): 5′-AGGTTAGTAAGTGGGT-3′; PEA15 ASO4 (ASO ID: 1235183): 5′-GGTTTAGAGTGCAAC-3′; PEA15 ASO5 (ASO ID: 1235218): 5′-AGTTAGCACGGTCAGG-3′.

### Statistical analyses

A *P* value less than 0.05 indicated significance in all other statistical analysis, and all statistical tests were 2-tailed Student’s *t* test. All statistical analyses were carried out in the R language environment (https://www.r-project.org).

## Results

### Three proteomic subtypes of HCC

**Fig. 1.**
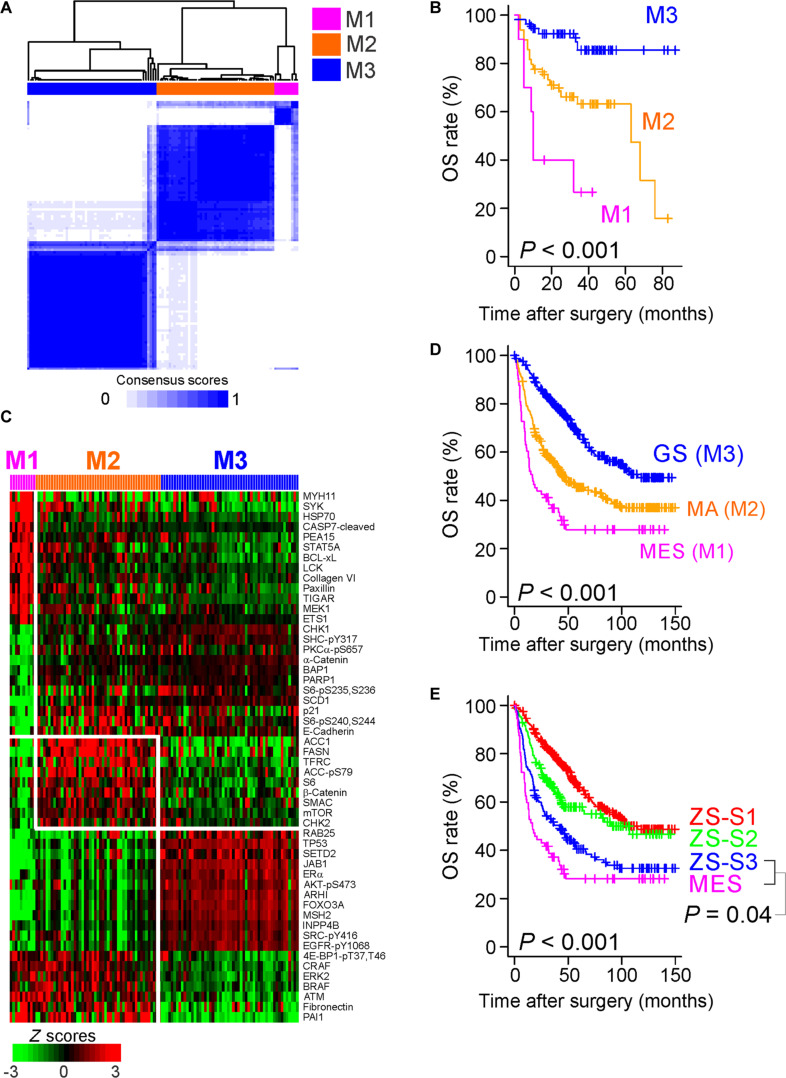
Prognostic significance of proteomic subtypes of hepatocellular carcinoma (HCC). (A) Hierarchical dendrogram and consensus score matrix of 3 subtypes identified by ConsensusClusterPlus (v. 3.12). The consensus heatmap displays the pairwise consensus scores (from 0 to 1) as indicated in the scale bar at the bottom. (B) Kaplan–Meier plot of overall survival (OS) in patients with the 3 HCC subtypes in the MD Anderson Cancer Center (MDACC) cohort (log-rank test). (C) Expression of 52 protein features in the 3 HCC subtypes in the MDACC cohort (*P* < 0.01, Student’s *t* test) and 1.3-fold difference between compared subtypes. Data (*z* scores) are presented in matrix format; each row represents an individual protein’s features, and each column represents one sample. Each cell in the matrix represents the expression level of a gene feature in an individual tissue sample. Red and green indicate relatively high and low expression levels, respectively, as indicated in the scale bar. (D) Kaplan–Meier plot of OS in the HCC patients in the pooled validation cohort (*n* = 690) stratified by the 3 HCC subtypes (log-rank test). (E) Kaplan–Meier plot of OS in the HCC patients in the pooled validation cohort stratified by Zhongshan Hospital subtype compared with the MES subtype (log-rank test). M1 to M3, MDACC subtypes 1 to 3; MES, mesenchymal subtype; MA, metabolically active subtype; GS, genome-stable subtype; ZS-S1 to ZS-S3, Zhongshan subtypes 1 to 3.

Unsupervised analysis of proteomic data revealed 3 subtypes of HCC with significant differences in prognosis (Fig. 1A and B and Fig. S1A). MDACC subtype 1 (M1) showed the poorest prognosis, while MDACC subtype 2 (M2) and subtype 3 (M3) showed moderate and best prognosis, respectively (Fig. 1B). We next identified 52 proteomic features specific to subtypes (Fig. 1C). M1 was characterized by high expression of myosin heavy chain 11 (MYH11), signal transducer and activator of transcription 5A (STAT5A), lymphocyte cell-specific protein-tyrosine kinase (LCK), mitogen-activated protein kinase/extracellular signal-regulated kinase (ERK) kinase 1 (MEK1), and PEA15. Furthermore, HCCs in this cluster had low expression of α-catenin and E-cadherin, indicating a transition from epithelial to mesenchymal characteristics. In comparison, the tumors in M2 had high expression of metabolic proteins, including acetyl-CoA (coenzyme A) carboxylase (ACC; ACACA and ACACB), fatty acid synthase (FASN), and transferrin receptor (TFRC). Notably, M3 tumors exhibited high expression of genome stability proteins, such as tumor protein TP53, MutS homolog 2 (MSH2), and SET domain-containing 2, histone lysine methyltransferase (SETD2), suggesting that these tumors had high genome stability. Accordingly, M1 was renamed the mesenchymal (MES) subtype of HCC, M2 the metabolically active (MA) subtype, and M3 the genome-stable (GS) subtype.

We sought to validate these 3 proteomic subtypes in the validation cohort of HCC patients from TCGA project (*n* = 184), in which the same RPPA platform was employed. Stratifying the patients in the TCGA cohort by their proteomic profiles (Fig. S1B) revealed highly consistent protein expression patterns in both cohorts (Fig. S1C), indicating that HCC proteomic traits are consistent across patient cohorts. We also generated mRNA expression data for the majority of the patients (*n* = 110) in the MDACC cohort and identified mRNAs whose expression was significantly associated with the 3 proteomic subtypes (Fig. S2A). To further validate the prognostic significance of 3 proteomic subtypes, we applied the mRNA signatures to pooled transcriptomic data from 5 independent HCC patient cohorts. Consistent with the MDACC cohort, the prognoses for the 3 HCC subtypes differed markedly in the pooled cohort (Fig. 1D).

We next compared our HCC subtypes with 3 previously identified proteomic subtypes at Zhongshan Hospital in Shanghai, China (ZS-S1, ZS-S2, and ZS-S3) [24]. We found that the GS subtype closely resembled ZS-S1, and the MES subtype was a subset of ZS-S3, the subtype with the poorest prognoses (Fig. S2B). Notably, the prognosis for the MES subtype was worse than that for ZS-S3 (Fig. 1E), suggesting that the MES subtype represents a potential proteomic subtype that has not been previously acknowledged. Consistent with our proteomic analysis (Fig. 1) showing high expression of genome stability proteins, the TCGA cohort revealed that the GS subtype had markedly fewer copy number alterations than the other subtypes (*P* < 0.001), whereas the mutation burden showed only marginal differences (Fig. S2C and D).

### *PEA15* was amplified in the MES subtype of HCC, associated with a poor prognosis for HCC, and indispensable for the proliferation and migration of HCC cells

**Fig. 2.**
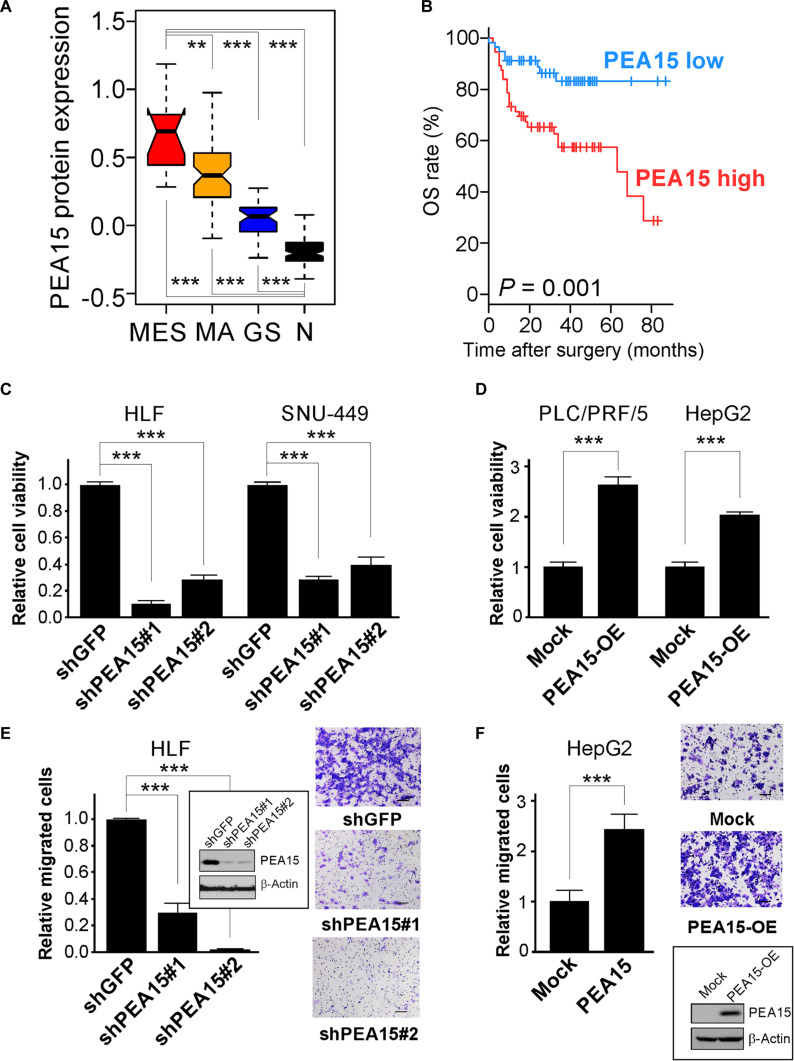
Proliferation and apoptosis adaptor protein 15 (PEA15) is accountable for poor prognosis for hepatocellular carcinoma (HCC) and necessary for the proliferation and migration of HCC cells. (A) Box plot of PEA15 protein expression in the 3 subtypes of HCC and surrounding normal tissue (N, Student’s *t* test). The horizontal black line within each box indicates the mean, the boundaries of each box indicate the 25th to 75th percentiles, and the whiskers above and below each box indicate the 10th and 90th percentiles. (B) Kaplan–Meier plot of overall survival (OS) in HCC patients in the MD Anderson Cancer Center (MDACC) cohort. The patients were stratified according to the expression of PEA15 protein (median cutoff; log-rank test). (C and D) Cell viability assay results for HCC cells with depletion or overexpression of PEA15. Depletion of PEA15 with shRNA specific to PEA15 (shPEA15-1 and shPEA15-2) significantly reduced the proliferation of mesenchymal cells (C). Expression of exogenous PEA15 significantly increased the proliferation of epithelial cells (D) (Student’s *t* test). (E and F) Cell migration assay results for HCC cells with depletion or overexpression of PEA15. Depletion of PEA15 with shPEA15 significantly reduced the migration of HLF cells (E). Expression of exogenous PEA15 significantly increased the migration of HepG2 cells (F). The whiskers indicate standard error. **P < 0.01, ***P < 0.001. MES, mesenchymal subtype; MA, metabolically active subtype; GS, genome-stable subtype; N, adjacent normal liver tissues; shGFP, shRNA specific to green fluorescent protein; shPEA15, short hairpin RNA for PEA15; PEA15-OE, PEA15 overexpression.

Among proteins exhibiting up-regulated expression in the MES subtype, PEA15 emerged as a particularly compelling candidate. Survival analysis demonstrated that both total PEA15 and its phosphorylated form (PEA15-pS116) were among the proteins most strongly associated with poor overall survival in HCC, with hazard ratios (HRs) exceeding 3 in the MDACC cohort (Fig. S3A). Given the report that PEA15 function might depend on phosphorylation status [38], we further examined this modification in our cohort. Both phosphorylated PEA15-pS116 and total PEA15 levels were significantly associated with poor survival (HR = 4.27, *P* < 0.001; HR = 3.16, *P* = 0.004, respectively), whereas the phospho-to-total ratio was not prognostic (HR = 1.34, *P* = 0.416). These findings suggest that phosphorylation at S116, although common in HCC, does not provide additional prognostic value beyond total PEA15 expression. PEA15 also displayed markedly cancer- and subtype-specific expression patterns, evident in its significantly higher expression in HCCs compared to adjacent normal tissues (Fig. S3B), along with a significant association of expression levels with the 3 HCC subtypes (Fig. 2A). Furthermore, high expression of PEA15 protein in the MDACC cohort was significantly associated with poor survival of HCC patients (*P* = 0.001; log-rank test) (Fig. 2B). To determine whether this prognostic effect was independent of established clinicopathologic factors, we conducted univariate and multivariate Cox regression analyses in the Zhongshan cohort (Table S2). After adjustment for sex, age, cirrhosis, hepatitis B core antibody (HBcAb) status, tumor size, tumor encapsulation, tumor number, nodal status, and α-fetoprotein level, high PEA15 expression remained an independent predictor of poor overall survival (HR = 1.66, 95% CI = 1.08 to 2.55, *P* = 0.018). These results underscore that the adverse prognostic effect of PEA15 expression is not attributable to confounding by known clinical factors. This association was consistent with data from the TCGA cohort, where PEA15 expression was significantly higher in the MES subtype compared to other subtypes (Fig. S3C), highlighting the crucial roles played by PEA15 in HCC. We further validated tumor-specific expression of PEA15 protein via WB analysis of the tissue lysates used in the RPPA experiment. Consistently, PEA15 expression was markedly higher in tumors than in adjacent normal tissues (Fig. S3D). Notably, PEA15 was also found to be highly phosphorylated in HCC tumors. HCC cell lines are largely divided into mesenchymal and epithelial cells [39]. In agreement with our findings in primary HCCs, mesenchymal HCC cell lines exhibited significantly higher PEA15 expression than epithelial HCC cell lines (Fig. S3E and F).

To explore molecular mechanisms governing increased expression of PEA15 in HCCs, we carried out several correlation analyses with proteomic and genomic data. These analyses revealed a strong correlation between PEA15 mRNA and protein expression as well as between mRNA expression and copy number alterations (Fig. S4A to D). These findings suggest that *PEA15* is predominantly activated by gene amplification in HCCs. Moreover, analysis across multiple tumor types using copy number data from the TCGA pan-cancer project showed frequent amplification of *PEA15* in various additional cancer lineages, including bladder cancer, cholangiocarcinoma, lung cancer, and breast cancer (Fig. S4E), indicating *PEA15* as an important driver across multiple cancers. Of the cancers we examined, HCC exhibited the second highest frequency of *PEA15* amplification.

Given the high amplification of *PEA15* in HCCs and its significant association with poor survival in HCC patients, we determined whether *PEA15* is necessary for maintaining the tumorigenic phenotype of HCC cells. In agreement with its clinical association, depletion of PEA15 expression by shRNA in the human mesenchymal HCC cell lines HLF and SNU-449 significantly reduced cell proliferation (Fig. 2C). This reduction in proliferation was corroborated by significant decrease in colony formation in HLF cells upon PEA15 depletion (Fig. S5A). Conversely, ectopic expression of PEA15 in the epithelial HCC cell line PLC/PRF/5 and the hepatoblastoma cell line HepG2 significantly increased cell proliferation and colony formation (Fig. 2D and Fig. S5B). Furthermore, depletion of PEA15 significantly reduced migration of the mesenchymal HCC cell lines HLF, SNU-475, and FOCUS (Fig. 2E and Fig. S5C and D). Conversely, ectopic expression of PEA15 in HepG2 cells significantly increased their migration (Fig. 2F). Importantly, coexpression of VHL abrogated the promigratory effect of PEA15, while VHL overexpression alone suppressed migration (Fig. S5E), confirming that VHL functions downstream of PEA15 in regulating HCC cell motility. Taken together, these data strongly suggest that PEA15 is indispensable for the proliferation and migration of HCC cells, which may contribute to the poor prognosis for HCC of the MES subtype.

### PEA15 exerted regulatory control over HIF1A by suppressing VHL in HCC cells

**Fig. 3.**
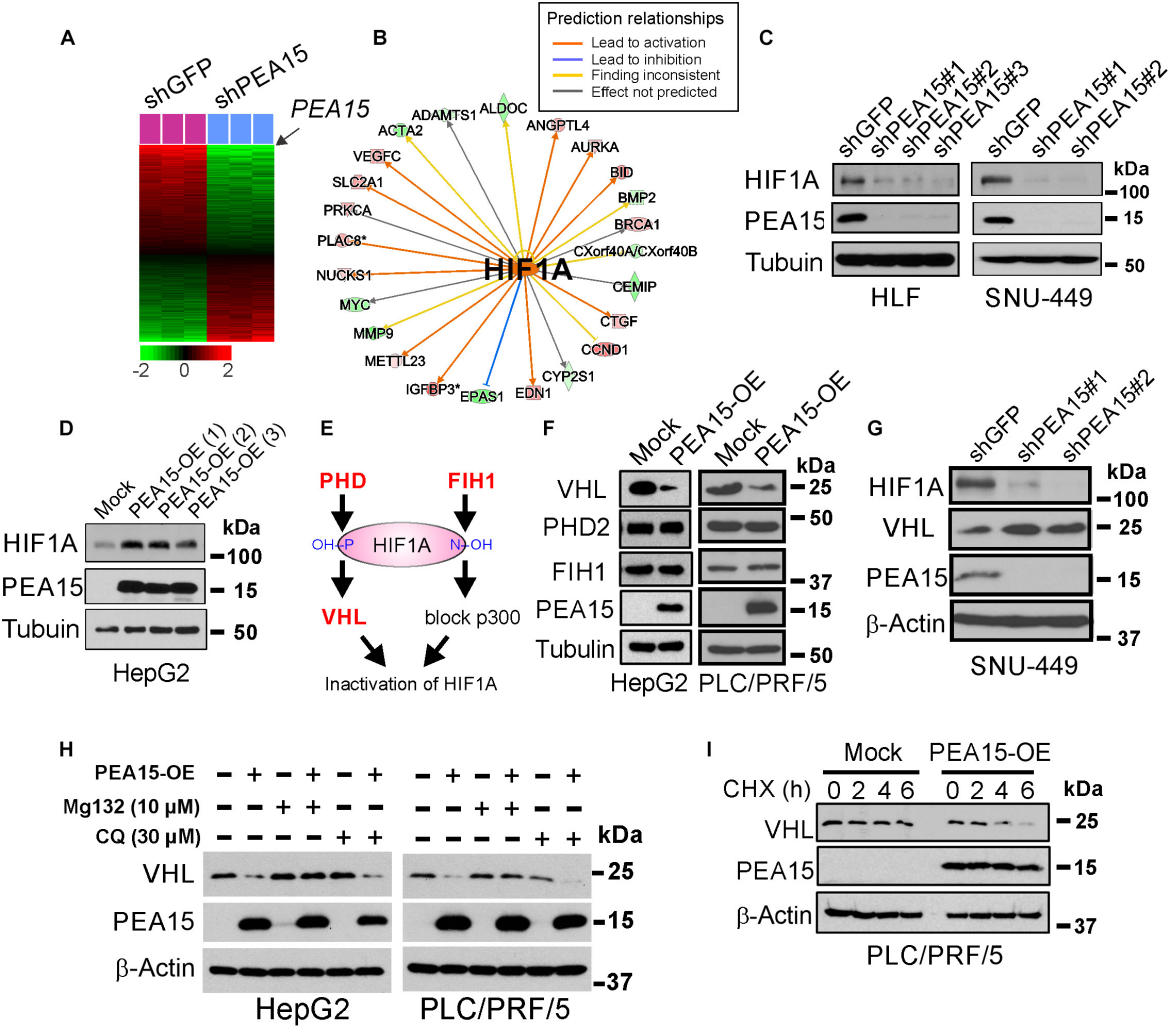
Proliferation and apoptosis adaptor protein 15 (PEA15) regulates hypoxia-inducible factor 1A (HIF1A) by down-regulating von Hippel–Lindau tumor suppressor (VHL) in hepatocellular carcinoma (HCC) cells. (A) Expression of 524 genes was significantly altered after depletion of PEA15 in FOCUS cells (*P* < 0.001). PEA15 was depleted 3 times. Data are presented in matrix format; each row represents an individual gene, and each column represents one sample. Each cell in the matrix represents the expression level of a gene feature in an individual tissue sample. Red and green indicate relatively high and low expression levels, respectively, as indicated in the log_2_-transformed scale bar. (B) Network analysis of PEA15-dependent genes revealed that many genes are downstream targets of HIF1A. The genes were color-coded according to the ratio of the expression of genes in cells treated with shGFP (control) to the expression of genes in cells treated with shPEA15. Red represents higher expression, and green represents lower expression in PEA15-active (control shGFP) cells. (C) Western blotting (WB) of PEA15 and HIF1A expression in PEA15-depleted (treated with shPEA15#1, shPEA15#2, and shPEA15#3) and control (treated with shGFP) HLF and SNU-449 cells. (D) WB of PEA15 and HIF1A expression in HepG2 cells with ectopic PEA15 overexpression. The numbers in parentheses indicate replicated experiments. (E) Schematic of HIF1A regulation by PHD, factor inhibiting HIF-1 (FIH1), and VHL. (F) WB of VHL, prolyl hydroxylase domain containing protein 2 (PHD2), and FIH1 expression in HepG2 and PLC/PRF/5 cells with ectopic PEA15 expression. PEA15 substantially reduced VHL expression in both cell lines. (G) WB of VHL and HIF1A expression in PEA15-depleted SNU-449 cells. VHL stability was substantially increased by silencing PEA15 expression. (H) WB of VHL and PEA15 expression after treatment with the proteinase inhibitor MG132 and the lysosomal inhibitor chloroquine (CQ) in HepG2 and PLC/PRF/5 cells. VHL degradation was reversed by MG132 but remained unaffected by CQ. (I) WB of endogenous VHL expression after treatment with cycloheximide (CHX) in PLC/PRF/5 cells with or without PEA15 expression. shPEA15, short hairpin RNA for PEA15; PEA15-OE, PEA15 overexpression.

To gain biological insight into the roles of *PEA15* in HCC development, we generated and analyzed gene expression data from PEA15-depleted FOCUS cells, identifying 524 genes whose expression was contingent on PEA15 (*P* < 0.001) (Fig. 3A). Notably, gene network analysis revealed that many of the PEA15-dependent genes were direct downstream targets of HIF1A (Fig. 3B and Table S3). In addition to HIF1A, SMAD3 and STAT3 were also predicted as upstream regulators of *PEA15*-dependent genes. To explore this, we examined their expression after PEA15 knockdown. Consistent with predictions, HIF1A was markedly down-regulated (Fig. 3C); however, SMAD3 was unexpectedly up-regulated, and STAT3 remained unchanged (Fig. S6A), indicating divergence from computational predictions. These results highlight HIF1A as the potential mediator of the PEA15-driven transcriptional program, whereas STAT3 and SMAD3 may act through context-dependent or compensatory mechanisms. In addition, HIF1A expression was significantly elevated in mesenchymal HCC cell lines compared to epithelial HCC cell lines (*P* = 0.001) (Fig. S6B and C) and significantly correlated with PEA15 expression in HCC cells (*r* = 0.83 and *P* = 0.001) (Fig. S6D). These results led us to hypothesize that PEA15 regulates HIF1A in HCC cells. In line with our hypothesis, HIF1A expression was substantially increased when ectopic PEA15 was expressed in HepG2 cells (Fig. 3D). Because PEA15 is crucial for the proliferation of HCC cells and positively regulates HIF1A stability, we next investigated whether PEA15 controls cell proliferation by regulating HIF1A. The ectopic expression of HIF1A successfully rescued the reduced proliferation of PEA15-depleted HLF and SNU-449 cells (Fig. S6E), strongly supporting the idea that PEA15’s control of cell proliferation is mediated through its regulation of HIF1A. To further assess whether PEA15–HIF1A signaling influences downstream transcriptional programs, we examined EMT-associated HIF1A target genes. PEA15 depletion consistently down-regulated SNAI1, VEGFC, SLC2A1, IGFBP3, PAI-1, and FN1 (Fig. S7A and B). These results support that PEA15 promotes HCC progression at least in part through activation of HIF1A-driven EMT and angiogenic pathways. Taken together, these data demonstrate that PEA15 is a potential regulator of HIF1A in HCC cells.

Given that HIF1A stability and activity are mainly regulated by PHDs [40,41], FIH1 [42,43], and VHL [17] (Fig. 3E), we examined the stability or expression of these 3 proteins after inducing ectopic expression of PEA15 in HepG2 and PLC/PRF/5 cells. Whereas the stability of PHD2 and FIH1 remained unchanged, VHL stability was substantially reduced upon ectopic PEA15 expression (Fig. 3F), suggesting that PEA15 regulates HIF1A stability through down-regulation of VHL. Consistent with this observation, depletion of PEA15 expression by shRNAs in SNU-449, HLF, and FOCUS cells increased VHL levels (Fig. 3G and Fig. S7C), providing further support for the notion that PEA15 regulates HIF1A through VHL in HCC cells. Importantly, PEA15 regulated VHL and HIF1A at the posttranscriptional level, as evidenced by a lack of change in their mRNA expression levels after PEA15 depletion in FOCUS cells (Fig. S7D). Moreover, treatment with the proteasome inhibitor MG132 abrogated PEA15-mediated reduction of VHL expression in HepG2 cells (Fig. 3H), suggesting that PEA15 regulates VHL predominantly via the proteasome. In contrast, treatment with the lysosomal inhibitor chloroquine failed to restore VHL levels in PEA15-overexpressing cells (Fig. 3H), indicating that lysosomal degradation does not contribute to PEA15-mediated VHL destabilization. In agreement with this, ectopic PEA15 expression substantially reduced the half-life of endogenous VHL in PLC/PRF/5 cells (Fig. 3I), suggesting that PEA15 increases the turnover rate for VHL in HCC cells. Given that PEA15 is phosphorylated at S104 and S116 in HCCs (Fig. S3D), we investigated whether PEA15 phosphorylation plays roles in the regulation of VHL stability and HCC cell proliferation. Overexpression of both PEA15 wild-type and PEA15 S104A/S116A phospho-null mutants down-regulated VHL and promoted HCC cell proliferation (Fig. S8), suggesting that PEA15 phosphorylation does not play a major role in the regulation of VHL, HIF1A stability, or proliferation of HCC cells.

### PEA15 disrupted VHL–HIF1A interaction and destabilized the VHL/ELOC/ELOB–cullin 2 E3 ligase complex

**Fig. 4.**
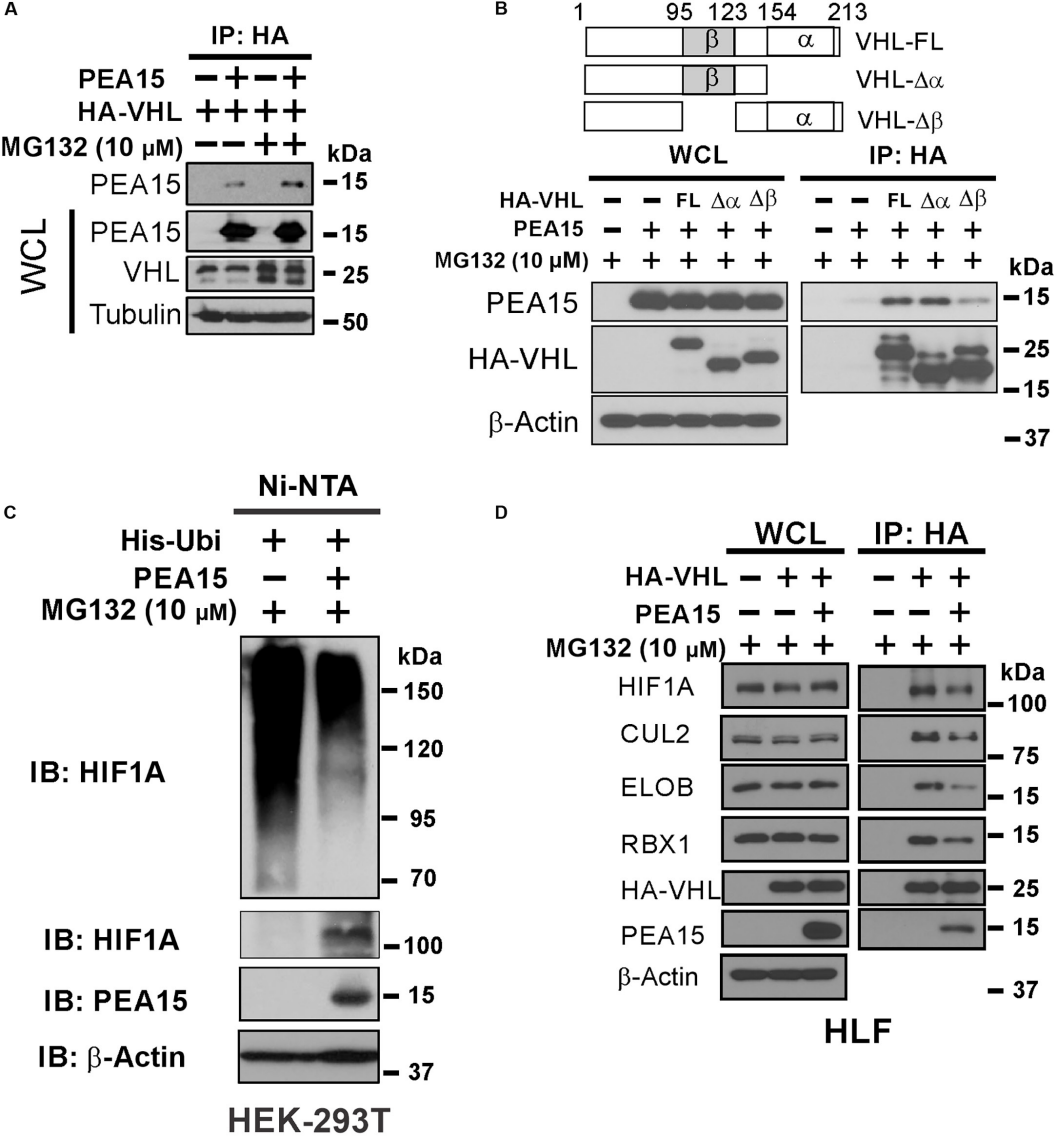
Proliferation and apoptosis adaptor protein 15 (PEA15) disrupts von Hippel–Lindau tumor suppressor (VHL)–hypoxia-inducible factor 1A (HIF1A) interaction and destabilizes the VHL/elongin C (ELOC)/elongin B (VCB)–cullin 2 (CUL2) complex. (A) Western blotting (WB) of PEA15 expression after immunoprecipitation (IP) of hemagglutinin (HA)-tagged VHL (HA-VHL) in HEK-293T cells transfected with expression vectors for PEA15 and HA-tagged VHL. (B) WB of fragmented VHL expression after coprecipitation with PEA15 in HEK-293T cells transfected with expression vectors containing VHL-FL, VHL-Δα, and VHL-Δβ and harvested 48 h after transfection. Cell extracts were immunoprecipitated with HA-tagged antibodies and immunoblotted with the indicated antibodies. (C) In vivo ubiquitination assay results for HEK-293T cells transfected with His-tagged ubiquitin (His-Ubi) and PEA15. Ubiquitinated hypoxia-inducible factor 1A (HIF1A) was precipitated with Ni-nitrilotriacetic acid (NTA) beads and probed with anti-HIF1A antibodies. (D) WB analysis performed to measure the stability of the VCB complex in HLF cells. The cells were transfected with expression vectors for PEA15 and HA-tagged VHL and harvested 48 h after transfection. Cell extracts were immunoprecipitated with anti-HA tag antibodies and immunoblotted with the indicated antibodies. His-Ubi, histidine-tagged ubiquitin; WCL, whole-cell lysates; IB, immunoblotting; VHL-FL, VHL full length; VHL-Δα, α-domain deleted VHL fragment; VHL-Δβ, β-domain deleted VHL fragment; VCB, VHL-ELOC-ELOB complex.

PEA15 co-immunoprecipitated with VHL (Fig. 4A), suggesting that the down-regulation of VHL is facilitated by the physical interaction between PEA15 and VHL. VHL, a component of the VHL/ELOC/ELOB (VCB)–cullin 2 (CUL2) E3 ligase complex, possesses 2 major interaction domains crucial for binding to ELOC (α-domain) and HIF1A (β-domain) [44,45]. The formation of the VCB–CUL2 complex through ELOC is critical for VHL’s stability [15,46,47]. To explore the molecular mechanism by which PEA15 regulates VHL, we performed an interaction assay with VHL fragments. Given that the stability of VHL was reduced following ectopic PEA15 expression, we anticipated that PEA15 would interrupt the interaction between the α-domain of VHL and ELOC, which is necessary for the stability of VHL [47]. However, to our surprise, PEA15 interacted with the β-domain of VHL (Fig. 4B). This interaction was confirmed through pull-down assays with purified recombinant proteins (Fig. S9A), suggesting that the increased stability of HIF1A results from the interruption of the HIF1A-VHL interaction, which is a crucial step for the degradation of HIF1A by the VCB–CUL2 E3 ligase complex. In agreement with this, ubiquitination of HIF1A was substantially reduced by PEA15 (Fig. 4C). Because reduced ubiquitination of HIF1A coincided with increased ubiquitination of VHL (Fig. S9B), we next tested whether PEA15 disrupts the integrity of the VCB–CUL2 complex, which is necessary for VHL stability and E3 ligase activity. A co-IP experiment demonstrated that PEA15 inhibited the interaction of VHL with other members of the VCB complex, such as CUL2, ELOB, and RBX1 (Fig. 4D).

**Fig. 5.**
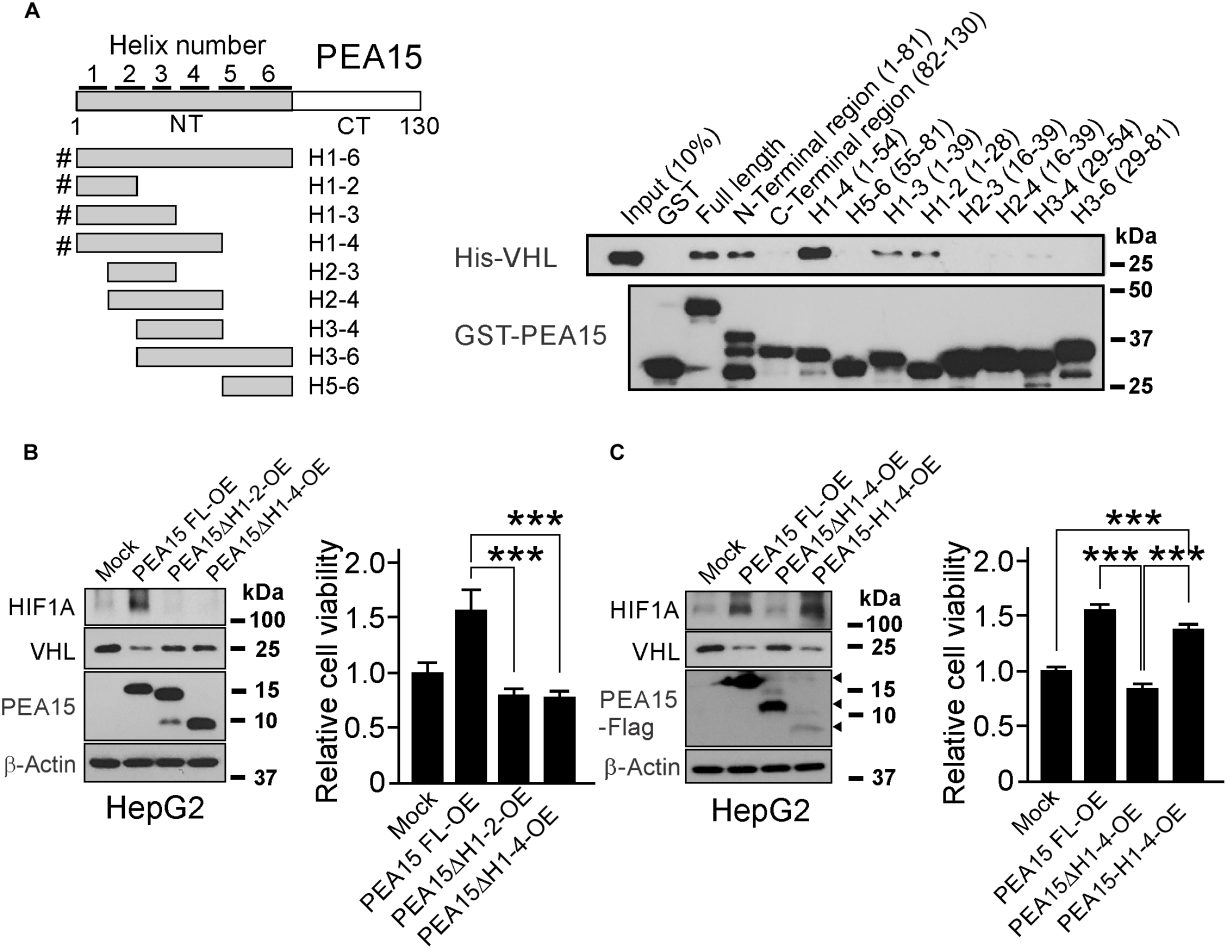
The N-terminal helices of proliferation and apoptosis adaptor protein 15 (PEA15) mediate von Hippel–Lindau tumor suppressor (VHL) binding and promote hypoxia-inducible factor 1A (HIF1A) stabilization and hepatocellular carcinoma (HCC) cell proliferation. (A) Results of a glutathione *S*-transferase (GST)–PEA15 pull-down assay with purified GST-fused PEA15 and recombinant VHL. Recombinant His-tagged VHL (His-VHL) was incubated with purified GST-fused fragmented PEA15 proteins as indicated, and a precipitated protein complex with Glutathione-Sepharose 4B beads was immunoblotted with anti-His probe antibodies. The H numbers in fragmented PEA15 indicate α-helix numbers in N-terminal regions of PEA15. The numbers in parentheses (except that for the input) indicate the residue numbers in PEA15. #PEA15 fragments that can interact with VHL. (B) Cell viability assay results for HepG2 cells with overexpression of fragmented PEA15. Deletion of the N-terminal region of PEA15 significantly abrogated the proliferation-promoting activity of PEA15 in these cells. Expression of fragmented PEA15 did not increase the stability of HIF1A. The whiskers indicate SE. (C) Cell viability assay results for HepG2 cells with overexpression of the N-terminal region of PEA15. The first 4 helices (H1–4) of PEA15 are sufficient to promote the proliferation of HepG2. Expression of PEA15-H1–4 increases the stability of HIF1A. The whiskers indicate SE. ***P < 0.001 (Student’s *t* test). His-VHL, histidine-tagged VHL; FL, full length; NT, N-terminal; CT, C-terminal; H, helix; PEA15 FL-OE, PEA15 full-length overexpression; PEA15ΔH1–2-OE, helix 1–2 deleted PEA15 overexpression; PEA15ΔH1–4-OE, helix 1–4 deleted PEA15 overexpression; PEA15H1–4-OE, PEA15 helix 1–4 fragment overexpression; GST-PEA15, GST-fused PEA15; PEA15-Flag, Flag-tagged PEA15.

To further dissect the molecular mechanism of PEA15-mediated regulation of VHL, we next performed an interaction assay with PEA15 fragments and found that VHL interacted with the N-terminal region of PEA15 (Fig. 5A). To further map the binding regions in the N-terminal region, we generated a series of truncated PEA15 fragments based on α-helical units in the N-terminal region domain (H1 to H6) and performed pull-down assays with VHL. It was found that VHL interacted with the first 4 α-helices at the N-terminal region (H1–4) but not with the remaining α-helices (H5–6) (Fig. 5A). Additional interaction assays showed that VHL interacted with the first 2 helices (H1–2) and first 3 helices (H1–3) but not with H3–4 or H3–6 at the N-terminal region, suggesting that the first 2 helices of the N-terminal region represent the minimal interaction region for VHL. Notably, N-terminal fragments lacking the first helix (H2–3 and H2–4) could not interact with VHL, indicating its essential role in the interaction with VHL. Moreover, VHL exhibited stronger interaction with H1–4 than it did with the full N-terminal helical region (NT) or full-length PEA15, suggesting that the C-terminal end of helical region H56 plays a negative regulatory function. Consistent with this finding, N-terminal deleted PEA15 (PEA15ΔH1–2 and PEA15ΔH1–4) failed to induce VHL degradation and increase the stability of HIF1A (Fig. 5B), demonstrating that the interaction of PEA15 with VHL through the N-terminal region of PEA15 is necessary for the regulation of VHL and HIF1A stability in HCC cells. In agreement with this, exogenously expressed N-terminal deleted PEA15 failed to stimulate the proliferation of HCC cells. Most importantly, the expression of the PEA15-H1–4 fragment induced VHL degradation, increased the stability of HIF1A, and stimulated the proliferation of HCC cells (Fig. 5C), suggesting that H1–4 alone is sufficient to increase the stability of HIF1A and promote cell proliferation. Taken together, these data suggest that PEA15 activates HIF1A by directly competing with interaction for the β-domain of VHL as well as by inducing degradation of VHL through destabilization of the VCB E3 complex.

### PEA15 was crucial for tumor growth

**Fig. 6.**
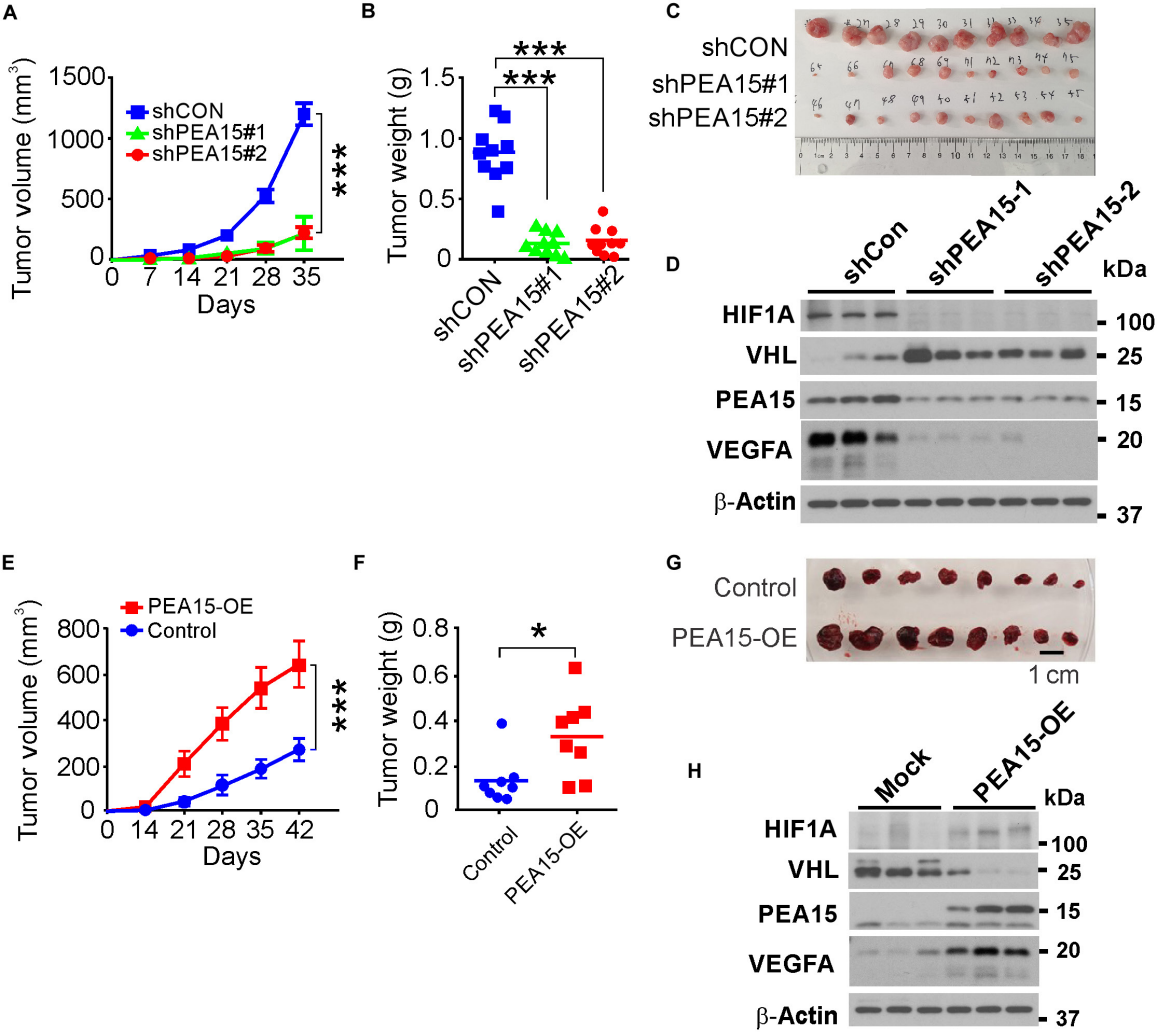
Proliferation and apoptosis adaptor protein 15 (PEA15) is necessary for hepatocellular carcinoma (HCC) tumor growth. (A) Growth of HLF tumors with and without depletion of PEA15 expression. HLF cells were xenografted subcutaneously in the flanks of mice, and the resulting tumor volumes were measured at the indicated time points for 5 weeks. (B) Weights of xenografted HLF tumors in mice. At 5 weeks after xenografting, the mice were euthanized, and their tumors were weighed (*n* = 10 per group). All data are presented as means ± standard error of the mean (SEM) (Student’s *t* test). (C) Representative images of xenografted HLF tumors from each group at week 5. (D) Western blotting (WB) analysis of the xenografted HLF tumors. (E) Growth of HepG2 tumors with and without PEA15 overexpression. HepG2 cells were xenografted subcutaneously in the flanks of mice, and the resulting tumor volumes were measured at the indicated time points for 6 weeks. (F) Weights of xenografted HepG2 tumors in mice. At 6 weeks after xenografting, the mice were euthanized, and their tumors were weighed (*n* = 8 per group). (G) Representative images of xenografted HepG2 tumors from each group at week 6. (H) WB analysis of the xenografted HepG2 tumors. All data are presented as means ± SEM (Student’s *t* test). *P < 0.05, ***P < 0.001 (Student’s *t* test). shPEA15, short hairpin RNA for PEA15; shCon, control shRNA; PEA15-OE, PEA15 overexpression.

As PEA15 expression was markedly associated with a poor prognosis for HCC and essential for the proliferation and migration of HCC cells, we next determined whether PEA15 is crucial for HCC growth in xenograft mouse models. We found that depletion of PEA15 significantly inhibited tumor growth in xenografted mice (Fig. 6A to C). Concurrently, there was an increase in VHL stability accompanied by a reduction in HIF1A level when PEA15 was depleted (Fig. 6D). Consistent with these results, overexpression of PEA15 markedly promoted tumor growth in the xenograft model, together with decreased VHL and elevated HIF1A levels (Fig. 6E to H). Collectively, these findings underscore the indispensable role of PEA15 in HCC growth, mediated through its regulation of VHL and HIF1A.

### Preclinical efficacy of PEA15 ASO therapy for HCC

**Fig. 7.**
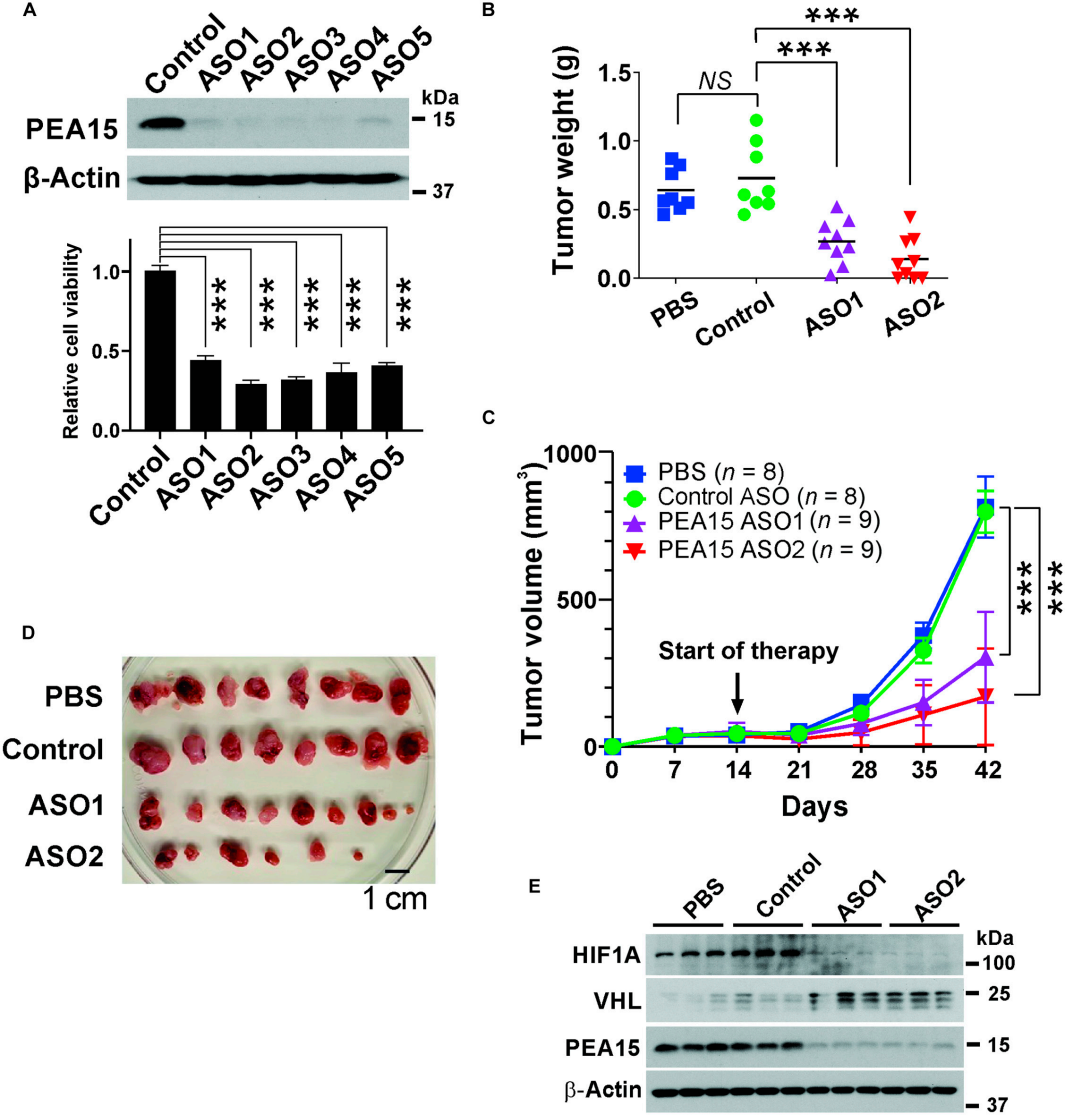
Therapeutic inhibition of proliferation and apoptosis adaptor protein 15 (PEA15) decreases tumor burden in hepatocellular carcinoma (HCC). (A) Cell viability assay results for HLF cells upon indicated antisense oligonucleotide (ASO) treatments (5 μM). HLF cells were treated with the indicated ASOs under free-transfection reagents. Expression of PEA15 was measured by Western blotting (WB) after 72 h of ASO treatment. (B) Weights of xenografted HLF tumors in mice. At 6 weeks after xenografting, the mice were euthanized, and their tumors were weighed (*n* = 8 to 9 per group). All data are presented as means ± standard error of the mean (SEM) (Student’s *t* test). (C) Growth of xenografted HLF tumors upon treatment of the ASOs. HLF cells were xenografted subcutaneously in the flanks of mice. After 14 d, HLF tumor-bearing mice were treated with the indicated ASO or vehicle control (50 mg/kg) 3 times a week, and the tumor volumes were measured at the indicated time points for 6 weeks. (D) Representative images of xenografted HLF tumors from each group at week 6. (E) WB analysis of the xenografted HLF tumors treated with control and PEA15-ASOs. ***P < 0.001 (Student’s *t* test).

While our findings highlight PEA15 as a compelling therapeutic target, targeting PEA15 poses considerable challenges because it functions as a regulatory interacting protein without enzymatic activity or easily accessible binding sites for small molecules, making the development of specific drugs against it a difficult endeavor. Given the inherent difficulty in targeting PEA15 due to its absence of easily druggable features, despite the significant inhibition of tumor growth observed upon PEA15 depletion (Fig. 6A and B), we sought to devise an alternative therapeutic approach for treating HCC. To do this, we screened and identified single-stranded ASOs that effectively target *PEA15* mRNA. ASOs function by hindering the expression of their target genes by hybridizing with the respective RNA sequence, which subsequently serves as a substrate for RNase H, leading to the degradation of the target mRNA [48]. By examining the efficacy of these ASOs in degrading *PEA15* mRNAs in HCC cells, we found that several ASOs significantly lowered PEA15 protein levels and inhibited the proliferation of HCC cells (Fig. 7A). Our investigation then extended to an in vivo context using 2 distinct PEA15-ASOs in HLF xenograft models. Remarkably, similar to the outcomes observed with PEA15 depletion, mice bearing HLF xenograft tumors and treated with PEA15-ASOs exhibited a significant reduction in tumor burden during the initial month of treatment compared to control groups (Fig. 7B to D). Importantly, the 2 different PEA15-ASOs led to a substantial decrease in PEA15 protein levels in xenografted tumors. Consequently, there was a marked up-regulation of VHL expression and a concomitant down-regulation of HIF1A expression (Fig. 7E), strongly indicating that the inhibition of tumor growth is due to the intended on-target effects of the PEA15-ASOs. Taken together, our findings clearly demonstrate that PEA15 represents a highly promising target for the treatment of HCC, and this therapeutic effect can be achieved through the use of ASOs.

## Discussion

**Fig. 8.**
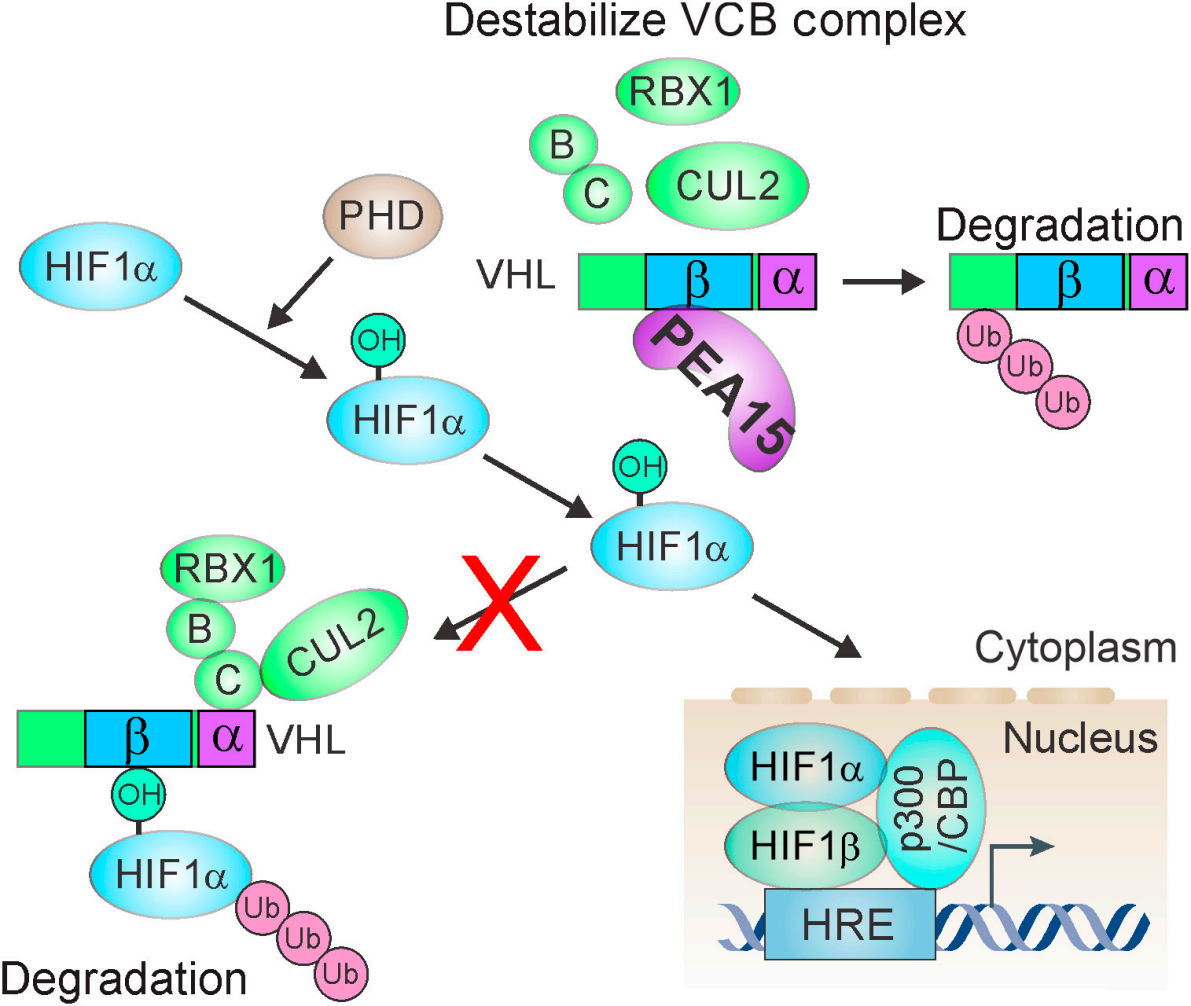
Schematic diagram of proliferation and apoptosis adaptor protein 15 (PEA15)-mediated regulation of von Hippel–Lindau tumor suppressor (VHL) and hypoxia-inducible factor 1A (HIF1A) stability in hepatocellular carcinoma (HCC) cells. PEA15 binds to VHL to inhibit the interaction between VHL and HIF1A and disrupt the stability of the VCB–CUL2 complex. B, elongin B; C, elongin C; VCB, VHL–elongin C–elongin B; HRE, hypoxia response element.

In the current study, we found that PEA15 was a novel regulator of the VHL/HIF1A axis by analyzing proteomic data (Fig. 8), unveiling a previously unrecognized mechanism that drove HCC progression. Building on this discovery, our integrative molecular, cellular, and in vivo analyses established PEA15 as a critical oncogenic driver with important biological and therapeutic implications.

Proteomic profiling of HCC tumors identified 3 distinct proteomic subtypes (MES, MA, and GS), which were significantly associated with clinical outcomes. Among these subtypes, MES exhibited the poorest prognosis with a high degree of EMT characteristics. Further analysis identified *PEA15* as a potential oncogenic driver of MES subtype. *PEA15*’s role in cancer has been controversial, with previous studies suggesting that it might act as either a tumor suppressor or promoter depending on the specific cellular context [49–53]. However, our data clearly showed that in HCC, *PEA15* functioned as a potent oncogene, promoting tumor aggressiveness. This oncogenic role was demonstrated by the amplification of *PEA15* in numerous tumor types, including HCC, and its strong association with poor clinical outcomes, as well as its indispensable roles in tumor growth and migration. Importantly, multivariate analysis in the Zhongshan cohort confirmed that high *PEA15* expression was an independent predictor of overall survival after adjustment for major clinicopathologic factors. Although phosphorylation of PEA15 was frequent in HCC tumors, it did not confer additional prognostic value beyond total PEA15 expression.

The most interesting findings of the current study was the molecular function of PEA15 as an upstream regulator of the VHL/HIF1A axis in HCC. The regulation of HIF1A is critical in various cancers as reflected in its contribution to the regulation of angiogenesis, the promotion of metastasis, and the resistance to therapy [11,12]. In normal physiological conditions, VHL, which is part of the VCB–CUL2 E3 ubiquitin ligase complex, targets HIF1A for proteasomal degradation under normoxic conditions [15–17]. Mutations in *VHL* are common in several cancers, particularly renal cell carcinoma, leading to uncontrolled HIF1A activity and tumor progression [15,54]. However, while HIF1A is frequently up-regulated in HCC [13,14], *VHL* mutations are exceedingly rare [18], leaving the mechanism of HIF1A activation largely unexplored until now. Our study revealed that PEA15 played a central role in HIF1A regulation by directly inhibiting the function of VHL. Specifically, PEA15 competed with HIF1A for binding to the β-domain of VHL, preventing the interaction with the VCB–CUL2 complex, which is necessary for HIF1A degradation. Additionally, PEA15 appeared to destabilize VHL itself likely by disrupting its interactions with other members of the VCB–CUL2 complex. Loss of VHL interaction with ELOB/C is known to compromise the stability of VHL [47], which in turn could explain the reduced VHL stability and the consequent increase in HIF1A accumulation observed in our study. One possible explanation for the destabilization of the VCB–CUL2 complex is that PEA15 bindings altered the conformational arrangement of VHL, thereby hindering its proper interaction with ELOB/C, leading to complex disassembly and VHL degradation. This mechanism of VHL inhibition by PEA15 resulted in the sustained HIF1A activation, promoting tumor growth and enhancing migration in HCC cells. Elevated HIF1A levels are generally associated with poor prognosis in HCC [13,14], and our study provided the mechanistic insight into how this activation occurs in the absence of *VHL* mutations. This finding positioned *PEA15* as a key player in HCC oncogenesis and highlighted its potential as a therapeutic target.

Another interesting finding was the potential of *PEA15* as a therapeutic target for HCC. Given its indispensable roles in promoting tumor growth and migration through the regulation of VHL/HIF1A pathway, targeting *PEA15* could provide a potential approach to battle HCC, particularly in patients with the MES subtype with the poorest clinical outcomes. Targeting PEA15 may not only reduce HIF1A activity but also restore VHL function, thereby attenuating the tumor-promoting effects of hypoxia-responsive genes. However, developing therapeutics to target PEA15 presents a considerable challenge. PEA15 functions as a regulatory protein and lacks the traditional enzymatic activity or accessible binding pockets typically targeted by small-molecule inhibitors. To overcome this, we explored the potential of ASOs as a strategy to inhibit *PEA15* mRNA expression. Our preclinical experiments demonstrated that PEA15-ASOs effectively reduced PEA15 protein levels in HCC cells, leading to significant reductions in cell proliferation. In vivo studies using xenograft mouse models provided further evidence of the therapeutic potential of targeting *PEA15*. Mice treated with PEA15-ASOs exhibited a marked reduction in tumor burden, accompanied by increased VHL and reduced HIF1A expression. These findings suggest that ASOs targeting *PEA15* could offer a viable therapeutic approach for patients with HCC, particularly those with the MES subtype.

Our study identified *PEA15* as a potential oncogene in HCC, and this finding opens opportunities for therapeutic intervention. Given the limitations of current therapies for advanced HCC, including the modest efficacy of multikinase inhibitors such as sorafenib and lenvatinib and the limited response to ICIs in HCC [2–9,55], targeting *PEA15* may offer a potential therapeutic strategy. By targeting the VHL/HIF1A pathway through PEA15 inhibition, we may be able to widen the therapeutic landscape that will eventually increase overall response to the therapies. Combining PEA15-ASOs with multikinase inhibitors or ICIs could result in potential synergistic effects, improving treatment responses of HCC patients. Additionally, PEA15 could serve as a valuable biomarker for patient stratification, allowing for the identification of individuals most likely to benefit from *PEA15*-targeted therapies. Although our study focused primarily on the role of PEA15 in regulating the VHL/HIF1A axis, it is likely that PEA15 has additional functions in HCC and other cancers. Gene network analysis suggested that PEA15 may regulate multiple downstream pathways, which could further contribute to tumor progression and resistance to therapy. Future studies should explore these additional roles of PEA15 to fully elucidate its contributions to cancer biology and identify other potential therapeutic targets within the PEA15-regulated network.

This study had several limitations. First, while our data clearly demonstrated that PEA15 disrupts the stability of the VCB–CUL2 complex and promotes VHL degradation, the precise structural and biochemical mechanism underlying this effect remains unknown. Further structural biology studies will be necessary to delineate how PEA15 binding perturbs complex integrity. Second, the functional validation of PEA15 was largely conducted in xenograft models using immunodeficient mice. These models did not capture the full spectrum of tumor–immune interactions, limiting our ability to assess the role of PEA15 in context of immune microenvironment of HCC. Third, although PEA15-targeted ASO therapy showed strong preclinical efficacy, its safety, pharmacokinetics, and delivery efficiency in humans remain untested, and additional translational studies are needed. Finally, while we focused primarily on the VHL/HIF1A axis, gene network analyses indicated that PEA15 may regulate multiple additional downstream pathways that were not fully explored in this study.

## Conclusion

In conclusion, our study provides critical insights into the role of *PEA15* as an oncogene in HCC, demonstrating its regulation of the VHL/HIF1A pathway and its impact on tumor growth and metastasis. *PEA15*’s amplification in HCC, particularly in the MES subtype, associates strongly with poor prognosis, making it an attractive target for therapeutic intervention. Our preclinical studies with PEA15-ASOs offer promising results, highlighting the potential of ASO-based therapies to inhibit *PEA15* and reduce tumor burden in HCC. These findings pave the way for the development of *PEA15*-targeted therapies and open possibilities for combination treatments that address the complex molecular landscape of HCC. Future research should continue to explore PEA15’s broader role in cancer and its potential as a therapeutic target across different cancer types.

## Ethical Approval

The MDACC Institutional Review Board (LAB09-0687-MDACC) approved this study with a waiver of consent and authorization under the Health Insurance Portability and Accountability Act Privacy Rule [45 CFR 164.512(i)].

## Data Availability

The datasets generated and analyzed during this study are publicly accessible. mRNA expression data from 110 tumors in the MDACC cohort can be found in the NCBI GEO database under accession number GSE134568. Gene expression data from various HCC patient cohorts are also available through the NCBI GEO database (GSE4024, GSE9843, GSE16757, GSE36376, and GSE54236) and the National Omics Data Encyclopedia (accession number OEP000321).
