# Supplementary material for: Proliferation and Apoptosis Adaptor Protein 15 (PEA15), a Potential Oncogenic Regulator of VHL and HIF1A Identified through Proteomic Analysis in Hepatocellular Carcinoma
Source: Cancer Commun (Lond). 2026 Apr 8;46:0020. doi: 10.34133/cancomm.0020 (PMC13058230; doi:10.34133/cancomm.0020)
Supplement: Supplementary 1 — Figs. S1 to S8 Tables S1 to S3 [file cancomm.0020.f1.zip › CANCOMM-D-25-00163.Supplementary.Clean.doc]

**Supplementary DATA**

**PEA15, a potential oncogenic regulator of VHL and HIF1A identified through proteomic analysis in hepatocellular carcinoma**

**Yun Seong Jeong, Ji-Hyun Shin, Soo Mi Kim, Bo Hwa Sohn, Sun Young Yim, Ji-Hoon Kim, Jae Jun Shim, Sung Hwan Lee, Yun Shin Chun, Sunyoung S Lee,** Hui Dai, **Ahmed Kaseb,** Koo Jeong Kang, **Holger K. Eltzschig,** A. Robert MacLeod, Xiaolin Luo, Alexey Revenko, Youngsoo Kim, **& Ju-Seog Lee**

**Supplementary Figures**

**Supplementary Figure S1. Conserved proteomic tumor subtypes in an independent HCC patient cohort.**

**(A)** Consensus cumulative distribution function (CDF) plot and delta area (change in CDF area) plot from analysis using ConsensusClusterPlus (v. 3.12). Subgroups are identified based on RPPA data of MDACC cohort (n = 113) by K-means consensus clustering upon their abundance. Delta area curve of consensus clustering indicates the relative change in area under the CDF curve for each k compared with k – 1. k indicates the number of subtypes **(B)** Schematic of a prediction model of HCC tumors with RPPA. HCC samples in the test cohort were categorized into three subtypes according to the Bayesian probability of each predictor. Tumors in the TCGA cohort (n = 184) were first stratified according to the M3 signature. Tumors in M1/M2 were further stratified according to the M1/M2 signatures. **(C)** Heatmap of the expression of 52 protein features in the three subtypes of HCC in the MDACC and TCGA cohorts. Data are presented in matrix format; each row represents an individual protein features, and each column represents one sample. Each cell in the matrix represents the expression level of a gene feature in an individual tissue sample. Red and green indicate relatively high and low expression levels, respectively, as indicated in the scale bar.

Abbreviations: HCC, hepatocellular carcinoma; CDF, cumulative distribution function; MDACC, MD Anderson Cancer Center; TCGA, The Cancer Genome Atlas; M1-3, MDACC subtypes 1-3; MES, mesenchymal subtype; MA, metabolically active subtype; GS, genome-stable subtype

**Supplementary Figure S2. Proteomic subtypes in HCC.**

**(A)** The gene expression (z-score) signatures specific to the three proteomic HCC subtypes in the MDACC cohort (n = 110). **(B)** Sankey diagram of a pair-wise comparison in the pooled validation cohort (n = 690). The MDACC subtypes are displayed in the left half, and the matched subtypes from the Zhongshan Hospital study are displayed in the right half. The ribbons indicate matched samples in the two classifications. **(C and D)** Association of proteomic HCC subtypes with copy-number alterations (C) and mutation burden (D) in the TCGA cohort. The genomic stability of each proteomic subtype was assessed according to mutation numbers and copy-number alterations. Copy-number alteration represents the fraction of the genome with altered copy numbers defined using GISTIC2 (Student t-test). In the box plots, the horizontal black line within each box indicates the mean, the boundaries of the boxes indicate the 25th to 75th percentiles, and the whiskers above and below the boxes indicate the 10th and 90th percentiles. ****P* < 0.001

Abbreviations: HCC, hepatocellular carcinoma; MES, mesenchymal subtype; MA, metabolically active subtype; GS, genome-stable subtype; ZS-S1-3, Zhongshan subtypes 1-3; MDACC, MD Anderson Cancer Center; TCGA, The Cancer Genome Atlas; GISTIC2, genomic identification of significant targets in cancer version 2.

**Supplementary Figure S3. Prognostic significance of PEA15 and its expression in HCC.**

**(A)** Forest plot depicting hazard ratios (HR) with 95% confidence intervals (CI) and corresponding P-values for MES-subtype protein features associated with overall survival in HCC. Proteins with HR > 1 were linked to poor prognosis, whereas HR < 1 suggested potential protective effects. PEA15 and PEA15-pS116 emerged as strong predictors of poor survival. The phosphorylation-to-total protein ratio of PEA15 was not a significant prognostic factor. **(B** and **C)** Box plots of PEA15 protein expression in HCC and adjacent normal liver tissue (N) samples in the MDACC cohort (E) and in the three proteomic subtypes of HCC in the TCGA cohort (F). **(D)** WB of the expression of PEA15 protein and PEA15 phosphorylated at S116 and S104 in HCC (T) and adjacent normal tissue (N) samples in the MDACC cohort (Student t-test). **(E)** WB of PEA15 protein expression in mesenchymal and epithelial HCC cell lines. *Note: HepG2 is of hepatoblastoma origin but displays epithelial characteristics. (**F**) Box plot of PEA15 expression in mesenchymal and epithelial HCC cells. PEA15 expression was normalized according to beta-actin expression (Student t-test). The horizontal black line within each box indicates the mean, the boundaries of the boxes indicate the 25th to 75th percentiles, and the whiskers above and below the boxes indicate the 10th and 90th percentiles. ****P* < 0.001

Abbreviations: HCC, hepatocellular carcinoma; HR, hazard ratio; CI, confidence intervals; MDACC, MD Anderson Cancer Center; TCGA, The Cancer Genome Atlas; MES, mesenchymal subtype; MA, metabolically active subtype; GS, genome-stable subtype; T, HCC tumors; N, adjacent normal liver tissues; WB, western blot; kDA, kilodalton.

**Supplementary Figure S4. Correlation of PEA15 mRNA, protein expression, DNA copy-number alterations in HCC patients.**

(A and B) Scatter plots of PEA15 protein and mRNA expression in the MDACC (A) and TCGA (B) cohorts (Pearson correlation coefficient). (C) Box plot of PEA15 expression according to amplification of its genomic loci in the TCGA cohort. The horizontal black line within each box indicates the mean, the boundaries of the boxes indicate the 25th to 75th percentiles, and the whiskers above and below the boxes indicate the 10th and 90th percentiles. (D) Scatter plot of *PEA15* mRNA expression and copy-number alterations in the TCGA cohort (Pearson correlation coefficient). (E) PEA15 copy-number alterations in multiple cancer types. The alteration frequency was assessed in TCGA data sets. PEA15 was amplified in the vast majority of the tumor types, with HCC having the second highest amplification frequency. ****P* < 0.001.

Abbreviations: HCC, hepatocellular carcinoma; MDACC, MD Anderson Cancer Center; TCGA, The Cancer Genome Atlas; DLBC, diffuse large B-cell lymphoma; PCPG, pheochromocytoma and paraganglioma; CS, clear cell carcinoma; ACC, adrenocortical carcinoma; GBM, glioblastoma multiforme; ccRCC, clear cell renal cell carcinoma; AML, acute myeloid leukemia; chRCC, chromophobe renal cell carcinoma.

**Supplementary Figure S5. Significant functional correlation of PEA15 with the growth and migration of HCC cells.**

**(A)** Colony formation assay results for HLF cells with depletion of PEA15. Depletion of PEA15 with shPEA15-1 and -2 significantly reduced colony formation (Student t-test). **(B)** Colony formation assay results for PLC/PRF/5 cells with expression of exogenous PEA15. The expression significantly increased the colony formation by these cells (Student t-test). **(C and D)** Cell migration assay results for SNU-475 (C) and FOCUS (D) cells with depletion of PEA15. The images on the right side of the panels show stained cells that migrated through the membrane. The whiskers indicate SE (Student t-test). **(E)** Cell migration assays in HepG2 cells with PEA15 and/or VHL overexpression. Quantification of migration showing that PEA15 enhanced cell migration, whereas VHL suppressed it. Importantly, co-expression of VHL abrogated the promigratory effect of PEA15. Scale bars indicate 100 µm. ****P* < 0.001.

Abbreviations: HCC, hepatocellular carcinoma; shGFP, short hairpin RNA for green flouresent protein; shPEA15, short hairpin RNA for PEA15; PEA15-OE, PEA15 overexpression; VHL-OE, VHL overexpression; NS, not significant; kDA, kilodalton.

**Supplementary Figure S6. PEA15 regulates HIF1A expression in HCC cells.**

**(A)** WB of SMAD3, STAT3, and PEA15 in HLF and SNU-449 cells after PEA15 knockdown; SMAD3 was upregulated, STAT3 unchanged. **(B)** WB of PEA15 and HIF1A expression in mesenchymal and epithelial HCC cell lines. *Note: HepG2 is of hepatoblastoma origin but displays epithelial characteristics.

**(C)** Box plot of HIF1A expression in HCC cells revealing higher expression of HIF1A in mesenchymal HCC cells (*P* = 0.001 by Student t-test). **(D)** Scatter plot of PEA15 and HIF1A expression in HCC cells revealing significant correlation of PEA15 with HIF1A (r = 0.83 and *P* = 0.001; Pearson correlation coefficient). PEA15 expression was normalized according to b-actin expression. The red and blue circles indicate mesenchymal and epithelial HCC cells, respectively. **(E)** Cell viability assay results for HLF and SNU-449 cells (*P* < 0.001; Student t-test). Expression of exogenous HIF1A rescued the PEA15 depletion phenotype. Cell growth was normalized to the shGFP control. Data are presented as the mean ± SEM of three independent experiments. ****P* < 0.001.

Abbreviations: HCC, hepatocellular carcinoma; WB, western blot; shGFP, short hairpin RNA for green flouresent protein; shPEA15, short hairpin RNA for PEA15; HIF1A-OE, HIF1A overexpression; NS, not significant; kDA, kilodalton.

**Supplementary Figure S7. PEA15 regulates EMT-related genes and VHL/HIF1A expression in HCC cells.**

**(A)** WB of SNAI1 expression in PEA15-depleted HLF and SNU-449 cells.

**(B)** Relative mRNA expression levels of EMT genes in PEA15-depleted FOCUS cells (Student t-test). **(C)** WB of VHL and HIF1A expression in PEA15-depleted HLF and SNU-449 cells. **(D)** Relative HIF1A and VHL mRNA expression levels in PEA15-depleted FOCUS cells (Student t-test). ****P* < 0.001.

Abbreviations: HCC, hepatocellular carcinoma; WB, western blot; shGFP, short hairpin RNA for green flouresent protein; shPEA15, short hairpin RNA for PEA15; NS, not significant; kDA, kilodalton.

**Supplementary Figure S8. PEA15 phosphorylation is not required for regulation of VHL or HIF1A expression.**

**(A)** WB of HIF1A and VHL expression in HepG2 and PLC/PRF/5 HCC cells with ectopic expression of wild-type and phospho-null (S104A and S116A) mutant PEA15. Both wild-type and mutant PEA15 substantially reduced VHL and increased HIF1A expression in both cell lines. **(B)** Cell viability assay results for HCC cells with overexpression of wild-type PEA15 and phospho-null mutant PEA15. Expression of both wild-type and mutant PEA15 significantly increased the growth of epithelial HCC cells (HepG2 and PLC/PRF/5; Student t-test). ***P* < 0.005, ****P* < 0.001.Abbreviations: HCC, hepatocellular carcinoma; WB, western blot; PEA15-OE, PEA15 overexpression; PEA15AA-OE, PEA15AA mutant overexpression; PEA15-pS104, PEA15 phosphorylated at the S104 residue ; PEA15-pS116, PEA15 phosphorylated at the S116 residue ;NS, not significant; kDA, kilodalton.

**Supplementary Figure S9. PEA15 regulates the ubiquitination of VHL.**

**(A)** In vitro GST pull-down assay results for the binding domain mapping of VHL interacting with PEA15. Full-length and fragmented GST-fused VHL were incubated with PEA15, precipitated with Glutathione-Sepharose 4B beads, and probed with anti-PEA15 antibodies. **(B)** In vivo ubiquitination assay results for HEK-293T cells transfected with His-Ubi, HA-VHL, and PEA15. Ubiquitinated VHL was precipitated with Ni-NTA beads and probed with anti-HA antibodies.

Abbreviations: HA, hemagglutinin tag ;His-Ubi, histidine-tagged ubiquitin; IB, immunoblotting; VHL FL, VHL full length; VHL-α, VHL α-domain fragment; VHL-β, VHL β-domain fragment; GST, Glutathione S-transferase; GST-VHL, GST-fused VHL; Ni-NTA, Ni-nitrilotriacetic acid; kDA, kilodalton.
